# DSGE Estimation Using Generalized Empirical Likelihood and Generalized Minimum Contrast

**DOI:** 10.3390/e27020141

**Published:** 2025-01-30

**Authors:** Gilberto Boaretto, Márcio Poletti Laurini

**Affiliations:** 1 Department of Economics, Rio de Janeiro State University (UERJ), Rio de Janeiro 20550-900, Brazil; gilbertoboaretto@hotmail.com; 2 Department of Economics, School of Economics, Business Administration and Accounting at Ribeirão Preto (FEA-RP/USP), University of São Paulo, Ribeirão Preto 14040-905, Brazil

**Keywords:** dynamic stochastic general equilibrium, method of moments, empirical likelihood, minimum contrast, minimum Hellinger distance

## Abstract

We investigate the performance of estimators of the generalized empirical likelihood and minimum contrast families in the estimation of dynamic stochastic general equilibrium models, with particular attention to the robustness properties under misspecification. From a Monte Carlo experiment, we found that (i) the empirical likelihood estimator—as well as its version with smoothed moment conditions—and Bayesian inference obtained, in that order, the best performances, including misspecification cases; (ii) continuous updating empirical likelihood, minimum Hellinger distance, exponential tilting estimators, and their smoothed versions exhibit intermediate comparative performance; (iii) the performance of exponentially tilted empirical likelihood, exponential tilting Hellinger distance, and their smoothed versions was seriously compromised by atypical estimates; (iv) smoothed and non-smoothed estimators exhibit very similar performances; and (v) the generalized method of moments, especially in the over-identified case, and maximum likelihood estimators performed worse than their competitors.

## 1. Introduction

Economic and statistical models, especially dynamic stochastic general equilibrium (DSGE) models, are susceptible to misspecification problems since they consist of simplifications (sometimes remarkably strong) of reality. The omission of relevant variables, incorrect functional forms, distributional assumptions, and incompleteness of systems of relations are common and often coincide in estimation and forecasting procedures [1]. Thus, we desire to employ estimation methods with good performance in the presence of misspecification. Here, robustness means insensitivity to small deviations from assumptions adopted, as defined by [2].

Misspecification problems can have global (non-local) or local natures. In moment-based estimation, global misspecification occurs when no parameter value is compatible with the moment restrictions, regardless of the sample size. Local misspecification occurs when these conditions are not satisfied in part of the sample, which disappears asymptotically [3]. See Appendix A for details on this and see Appendix B for a more detailed discussion of local and global misspecification in moment-based estimation. In likelihood-based estimation, consistency can be significantly affected by the validity of distributional assumptions; the quasi-maximum likelihood estimator may or may not be consistent for particular parameters of interest [4,5]. In this approach, we can consider this case as global misspecification. The authors of [6] and other papers introduce local misspecification through a nuisance parameter that contaminates the sample and whose effect disappears asymptotically. Depending on the estimation approach, both local and global misspecification problems are usually present in DSGE estimation.

In the current literature on DSGE modeling, we observe two distinct approaches, as highlighted by [7]. The first, which uses the likelihood principle, emerged in [8] and is based on approximations of the model’s police functions obtained by linearizing the equilibrium conditions around the steady state. This technique allows for evaluating the likelihood function using the Kalman filter or particle filter. The parameters are estimated either by classical inference maximizing the likelihood function (maximum likelihood—ML) or by Bayesian inference (BI) combining the likelihood function and prior distribution to obtain the parameters’ posterior distribution.

The moment-based approach, in turn, uses a set of moment conditions generated from the model’s first-order conditions (FOCs). The first works that employed GMM in DSGE estimation were [9,10,11]. Among the advantages of GMM estimation about ML estimation (and BI) are (i) to require fewer constraints for the data distribution and (ii) to require less computational capacity and, consequently, less estimation time [7,12]. Ref. [13] concluded that moment-based methods, more specifically GMM and the simulated method of moments (SMM), present better results than the ML estimator in terms of estimation speed and robustness to specification problems. Ref. [12] showed that GMM and SMM estimators obtained more accurate estimates for model parameters, even in small samples.

This paper aims to analyze the performance of moment-based estimators belonging to generalized empirical likelihood (GEL) and generalized minimum contrast (GMC) families in DSGE estimation, using GMM, ML, and BI as benchmarks. The generalized empirical likelihood (GEL) and generalized minimum contrast (GMC) methods generalize the use of moment methods when performing the nonparametric estimation of the process distribution, obtaining better properties in finite samples in terms of bias and some additional robustness properties. Thus, it is interesting to analyze whether the use of these methods can bring gains in the estimation of DSGE models since these models are commonly estimated from small samples and under relevant simplifications and restrictions.

In minimum contrast estimation, entropy measures, especially those rooted in Kullback–Leibler divergence, are often used to quantify the “distance” between an empirical distribution and a theoretical model. This approach is valuable when likelihood functions are complex or unspecified, allowing for the use of contrast functions like entropy to approximate them [14,15]. By minimizing the “contrast” (e.g., the divergence) between observed and model-based distributions, estimators can achieve a form of alignment that captures the underlying statistical structure with minimal assumptions.

Entropy concepts, particularly in generalized empirical likelihood (GEL), serve a dual role. They adjust likelihood estimates by introducing penalties for models that diverge from empirical data, thereby helping to align model-based probabilities more closely with observed data. GEL methods use entropy-based divergences, such as Kullback–Leibler divergence, to balance fit with the need for generalization, reducing overfitting by choosing model parameters that satisfy the given constraints [16]. This information-theoretic approach to estimation, encompassing methods like GEL and minimum contrast, emphasizes entropy’s role in making robust inferences that respect both model and data uncertainties.

Our paper extends other contributions to the employment of GEL/GMC estimators in the estimation of DSGE and economic models in general. Two moment-based estimators were considered in DSGE estimation by [7]. The author estimated a DSGE model using GMM and exponentially tilted empirical likelihood (ETEL), a GEL/GMC estimator. ETEL did not obtain results as close to true values as the GMM estimator, and, in addition, the ETEL results presented a high standard deviation. The two estimators presented difficulties in dealing with the identification of the utility function curvature parameter. The author encouraged the continuity of research in this field due to the advantages presented by empirical likelihood, such as (i) the direct use of the equilibrium conditions of the model since it is not necessary to compute the police function; (ii) the flexibility to assume distributions for the stochastic process of the economy; and (iii) the preservation of the nonlinear structure of the equilibrium conditions of the model.

Several applications of moment-based GEL/GMC estimators have been made in finance. Among the covered topics, we highlight the following: portfolio selection based on the empirical likelihood and Hellinger distance [17]; asset pricing models under misspecification and robustness analysis [18]; estimation of the discretized stochastic differential equation for interest rates [19]; dealing with hard assumptions of Black–Scholes option pricing [20]; obtaining risk measures of the risk of loss on a specific portfolio, such as value at risk and expected shortfall [21]; improvement in estimation precision for portfolio optimization [22]; and portfolio efficiency tests with conditioning information in the presence of data contamination [23]. These papers explored the good robustness and other properties of estimators belonging to the class of GEL/GMC estimators under misspecification and data contamination. They found promising results both in simulations and empirical applications.

Ref. [18] highlighted the possibilities of robustness analysis in the estimation of misspecified asset pricing models using GEL/GMC methods. Ref. [19] found that GEL/GMC estimators (mainly the ETEL) outperform the GMM, in terms of bias and mean squared error, in the estimation of stochastic differential equations for interest rates. Ref. [23] showed that GEL estimators perform better than the GMM in portfolio efficiency tests with conditioning information in the presence of data contamination, such as heavy tails and outliers. Ref. [24] applied adjusted empirical likelihood to make robust inferences about the Sharpe ratio in asset pricing and [25] analyzed the properties of the maximum empirical likelihood, maximum empirical exponential likelihood, and maximum log Euclidean likelihood estimators to estimate spatial autocorrelation models.

We deal with a real business cycle (RBC) model that can be considered the core of current DSGE models. We verify by means of Monte Carlo experiments if the studied estimators generate satisfactory results in terms of bias and variance measures in situations where the estimated model is correctly specified. We also emphasize the robustness analysis under both local and global misspecification situations. While in the correctly specified model productivity shocks follow a normal distribution, in misspecified models, we generate productivity shocks following a Student’s t-distribution or a normal distribution with the inclusion of single or several outliers.

The objective of this article is to analyze moment condition-based methods applied to the estimation of DSGE models, with a particular focus on extending the use of the generalized method of moments (GMM) to alternative approaches grounded in moment conditions. Specifically, this study evaluates the strengths and limitations of methods such as empirical likelihood and generalized minimum contrast, considering their performance under both correctly specified and misspecified model settings.

Among the main results of our paper for the estimation of a DSGE model are the following: (i) the empirical likelihood (EL) estimator, as well as its version with smoothed moment conditions (SEL), and Bayesian inference (BI) obtained, in this order, the best performances, even in misspecification cases; (ii) continuous updating empirical likelihood (CUE), minimum Hellinger distance (HD), exponential tilting (ET), and their smoothed versions presented intermediate comparative performance; (iii) ETEL, exponential tilting Hellinger distance (ETHD) estimators, and their smoothed versions were compromised by the occurrence of atypical estimates; (iv) smoothed and non-smoothed versions of the GEL/GMC estimators exhibited very similar performances; and (v) GMM, especially in the over-identified case, and ML estimators performed worse than their competitors.

These experiments show some cases of real problems that may affect the DSGE estimation. Thus, our study contributes to the still limited literature on the robust estimation of DSGE models. As these models have high importance in the analysis and conduct of economic policies, our study contributes some recommendations about using estimation methods in situations of both correct and incorrect specification.

This paper is structured in four additional sections besides this introduction and the Appendix. In Section 2, the economic model is presented. Section 3 presents the estimators and Monte Carlo design. Section 4 discusses the results. Section 5 concludes this paper. Lastly, the Appendix shows the derivation of the moment conditions used in the moment-based approach and the definitions of local and global misspecification.

## 2. Model

This section presents the economic model used in this paper. We will work with the “one consumer–one producer” version of the RBC model, similar to the model proposed by [26] and the standard stochastic growth model used by [12]. This version has a price vector (real wage and interest rate) and firm maximization problem. We consider a utility function in which consumption preferences are characterized by constant relative risk aversion and leisure (labor disutility) is linear, as defined in the model used by [12]. This model presents both the core components and estimation difficulties of the DSGE models, and at the same time, it has few parameters which facilitate the analysis of results.

Preferences

Suppose that the economy’s population is identical and that the representative consumer preferences are characterized by the instantaneous utility function

$$u\left(c_{t},n_{t}\right)=\frac{c_{t}^{1-\gamma }}{1-\gamma }+b\left(1-n_{t}\right),\;\;\gamma ,b>0$$

where $c_{t}$ is consumption in period *t*, $n_{t}$ is leisure time in period *t*, γ is the CRRA parameter, and *b* measures the disutility of labor (or utility of leisure). Time endowment and population size are constant and normalized to 1.

Production

Suppose there exists a single perishable good in the economy, $y_{t}$, whose production can be described by the function

(1) $$y_{t}=f\left(k_{t},n_{t},z_{t}\right)=z_{t}k_{t}^{\alpha }n_{t}^{1-\alpha }$$

where α∈(0,1) is a parameter, $k_{t}$ is the capital stock in period *t*, and $z_{t}$ is an exogenous productivity shock that occurred in *t*. Since this function is homogeneous of degree one, it exhibits constant returns to scale.

Laws of motion and feasibility

Consider a technological level described by

(2) $$\ln z_{t}=\rho \ln z_{t-1}+\varepsilon_{t},\;\;\varepsilon_{t}∼D\left(0,\sigma^{2}\right)$$

where ρ∈(−1,1) indicates that the technology follows a stationary process, and $\varepsilon_{t}$ is an independent and identically distributed (iid) shock following a generic distribution D with mean zero and variance $\sigma^{2}$.

Suppose that the capital stock evolves according to

(3) $$k_{t+1}=i_{t}+\left(1-\delta \right)k_{t},\;k_{0}>0$$

where δ∈[0,1] is the depreciation rate and $i_{t}$ is the investment in period *t*. In this way, the economy must respect the feasibility condition expressed by

(4) $$c_{t}+i_{t}=w_{t}n_{t}+r_{t}k_{t+1}+\pi_{t}$$

where $w_{t}$ is real wage, $r_{t}$ is the real interest rate, and $\pi_{t}$ is firm profit. Note that prices $w_{t}$ and $r_{t}$ are expressed in units of the consumption good.

Maximization problems, optimality conditions, and competitive equilibrium

In this economy, the representative consumer solves the following maximization problem

$$\max_{{\left\{c_{t},n_{t},k_{t+1}\right\}}_{t=0}^{\infty }}E_{0}\sum_{t=0}^{\infty }\beta^{t}u\left(c_{t},n_{t}\right)$$

subject to (1)–(4). The representative firm, in turn, solves the following static maximization problem

$$\max_{y_{t},n_{t},k_{t+1}}\pi_{t}$$

with $\pi_{t}=y_{t}-w_{t}n_{t}-r_{t}k_{t+1}$, subject to (1)–(3). The optimal choices of consumption and labor supply are given by the first-order conditions

(5) $$\begin{array}{c} c_{t}^{\gamma }=\frac{w_{t}}{b} \end{array}$$

(6) $$\begin{array}{c} c_{t}^{-\gamma }=\beta E_{t}\left[\left(1-\delta +r_{t+1}\right)c_{t+1}^{-\gamma }\right] \end{array}$$

which are the intratemporal relation between consumption and labor and the Euler equation for consumption, respectively. The firm’s first-order conditions are given by

$$\begin{array}{c} r_{t}=\alpha z_{t}k_{t}^{\alpha -1}n_{t}^{1-\alpha }\\ w_{t}=\left(1-\alpha \right)z_{t}k_{t}^{\alpha }n_{t}^{-\alpha }. \end{array}$$

The competitive equilibrium of this economy is given by the sequence of prices ${\left\{w_{t},r_{t}\right\}}_{t=0}^{\infty }$ and by the sequence of allocations ${\left\{c_{t},k_{t+1},n_{t}\right\}}_{t=0}^{\infty }$ such that, given prices, consumer utility and firm profit are maximized and goods, capital, and labor markets are clearing.

Steady state and policy functions

After obtaining steady-state values, the optimal allocation sequence is obtained from the policy functions. Ref. [27] points out that, even with the computational advancement, to obtain the policy functions, it is necessary to resort to some approximation when the model is more complicated and the dimension of state variables increases. From the log-linearized FOC equations, we proceed by writing the model in matrix form, relating all variables contemporaneously, as well as the lagged and leading values, and obtaining the policy functions [27]. Among the procedures that fulfill this task, [28,29] stand out. For further details, see [27,30,31].

## 3. Methodology

### 3.1. Estimators

#### 3.1.1. Generalized Method of Moments

Ref. [32] derived the generalized method of moments (GMM) estimator and demonstrated its large-sample properties. Let $h(x_{t},\theta_{0})$ be a vector of moment conditions where $\theta_{0}$ is a vector with the true parameters, and $x_{t}$ are random variables. In this case, we have $E[h\left(x_{t},\theta_{0}\right)]=0$. The sample mean of $h(x_{t},\theta_{0})$ generates the sample moment condition $g\left(x_{t},\theta \right)\equiv \frac{1}{T}\sum_{t=1}^{T}h\left(x_{t},\theta \right)$, where θ is a vector of unknown parameters belonging to parametric space Θ, and *T* indicates the sample size. From this, in the over-identified case, we define the GMM estimator by

$${\hat{\theta }}_{GMM}=\underset{\theta \in \Theta }{arg min}g{\left(x_{t},\theta \right)}^{\prime }Wg\left(x_{t},\theta \right)$$

where *W* is a positive-definite weighting matrix whose optimal form corresponds to the inverse of the asymptotic variance matrix [33]. However, the asymptotic variance matrix is a function of parameters and, therefore, must be estimated. Refs. [34,35] define heteroscedasticity and autocorrelation-consistent (HAC) estimators for asymptotic variance.

In this paper, we use the two-step GMM (2SGMM) estimator. Iterative and continuous updating versions presented optimization and convergence problems and, in general, did not prove to be computationally reliable in DSGE estimation. The 2SGMM procedure, initially proposed by [32], is based on an initial weighting matrix *W*, usually the identity matrix. From this first step, the HAC matrix $\hat{S}\left(\hat{\theta_{1}}\right)$ is calculated, where $\hat{\theta_{1}}$ is a parameter vector estimated in the first step. The next step starts using the matrix obtained in the first step, and ${\hat{\theta }}_{2}$ is obtained by the minimization of the GMM objective function. If the system is over-identified, that is, if the number of moment conditions is greater than the number of parameters, we can employ the J test proposed by [32], whose null hypothesis is a well-specified model (namely, valid moment conditions).

#### 3.1.2. Generalized Empirical Likelihood and Generalized Minimum Contrast

##### Generalized Empirical Likelihood (GEL) and EL, ET, and CUE Estimators

The generalized empirical likelihood (GEL) class, initially proposed by [36], is a unifying framework encompassing estimators with a common structure. These estimators are asymptotically equivalent to 2SGMM and have better higher-order asymptotic properties than the latter, as well as better performance in small-sample cases [37,38,39].

Let ρ(υ) be a function whose domain is a convex set Υ containing the zero. The GEL estimator is expressed by the saddle point problem

(7) $${\hat{\theta }}_{GEL}=\underset{\theta \in \Theta }{arg min}\underset{\lambda \in \Lambda }{\sup}\sum_{t=1}^{T}\rho \left(\lambda^{\prime }g\left(x_{t},\theta \right)\right)$$

where $\Lambda =\{\lambda :\lambda^{\prime }g\left(x_{t},\theta \right)\in \Upsilon \}$[37,40]. The choice of the function ρ(υ) defines the following estimators: (1) the empirical likelihood (EL) of [41,42,43]: ρ(υ)=ln(1−υ); (2) the exponential tilting (ET) of [44,45]: ρ(υ)=exp(υ); and (3) the GMM continuous updating (CUE) of [46]$:\rho \left(\upsilon \right)=-{\left(1+\upsilon \right)}^{2}/2$ [37,39,47].

Empirical likelihood is a nonparametric method that constructs a likelihood function directly from the data. Given a set of moment conditions, this method avoids assuming a parametric form for the likelihood and yields estimators with asymptotically normal distributions. One notable property of EL is its Bartlett correctability, which allows for the adjustment of confidence intervals to improve their higher-order accuracy. However, EL can be sensitive to model misspecification, particularly when the moment conditions involve unbounded functions, as this can lead to a loss of $\sqrt{n}$ consistency in estimation. Exponential tilting modifies the empirical likelihood framework by introducing exponential weights to observations. ET enhances robustness to model misspecification, maintaining $\sqrt{n}$ convergence rates even when the assumed model is not perfectly specified. However, unlike EL, ET is not Bartlett-correctable, limiting its ability to achieve higher-order accuracy in confidence intervals.

The generalized method of moments (GMM) continuous updating estimator (CUE) offers certain theoretical advantages, such as asymptotic efficiency under correct specification, by incorporating an optimal weighting matrix that depends on the parameter estimate θ. However, it is prone to several numerical instabilities, which can limit its practical implementation. These instabilities arise from the nonlinearity of the objective function, sensitivity to the choice of instruments, and properties of the weighting matrix, as discussed by [46].

##### Generalized Minimum Contrast (GMC) and Its Relationship with GEL

The GEL can be considered a special case of generalized minimum contrast (GMC) class of estimators that is a generalization of the ideas contained in [48,49]. Following [38], consider a general divergence function between two measures of probability, *P* and *Q*, expressed by

(8) $$D\left(P,Q\right)=\int \phi \left(\frac{dP}{dQ}\right)dQ$$

where ϕ(·) is a convex function. One of these probability measures usually follows a nonparametric data distribution, and the other corresponds to a statistical distribution associated with some model. Let $x_{t}\in R^{n}$ be iid random variables, where *n* is the number of random variables. Consider a model that follows the moment conditions $E\left[g\left(x_{t},\theta \right)\right]=\int g\left(x,\theta \right)d\mu =0,\;\theta \in \Theta \subset R^{q}$, where $g\in R^{q}$ is a known function, *q* is a number of parameters, and Θ is a parametric space. Let M be the set of all probability measures in $R^{n}$ and define

$$P=\underset{\theta \in \Theta }{⋃}\left\{P\in M:\int g(x_{t},\theta )dP=0\right\},$$

that is, P represents the set of all probability measures compatible with the moment restrictions. Model P is correctly specified only if it includes true probability measures μ. Thus, the GMC optimization problem is given by

(9) $$\underset{\theta \in \Theta }{\inf}\underset{P\in P}{\inf}D\left(P,\mu \right).$$

Following [37,50], the minimal contrast problem in (9) can be rewritten using a contrast function $h_{T}\left(p_{t}\right)$:

$${\hat{\theta }}_{MC}=\underset{\theta ,p_{t}}{arg min}\sum_{t=1}^{T}h_{T}\left(p_{t}\right)$$

Assuming [51] a family function of discrepancies given by

(10) $$h_{T}\left(p_{t}\right)=\frac{{\left[\gamma \left(\gamma +1\right)\right]}^{-1}{\left(Tp_{t}\right)}^{\gamma +1}-1}{T},$$

where γ is an indexing parameter, we obtain the estimators of the GEL family, which also belong to the GMC family, assigning specific values for γ: (1) EL: γ=0; (2) ET: γ=−1; and (3) CUE: γ=1 [37,47,50].

##### Computational Implementation of the GEL/GMC Estimators

After the algebraic development of the optimization problem, Ref. [38] arrives at the following result that defines the GMC estimator for the sample case

$${\hat{\theta }}_{GMC}=\left\{\underset{\theta \in \Theta }{arg min}\inf\frac{1}{T}\sum_{t=1}^{T}\phi \left(tp_{t}\right):\sum_{t=1}^{T}p_{t}=1;\;\sum_{t=1}^{T}p_{t}g\left(\theta ,x_{t}\right)=0\right\},$$

where ϕ is a convex function. A convenient analogue for computational implementation deriving from the duality theorem present in [52] is given by

(11) $${\hat{\theta }}_{GMC}=\underset{\theta \in \Theta }{arg min}\left\{\max_{\lambda ,\gamma \in R^{n}}\left[\lambda -\frac{1}{T}\sum_{t=1}^{T}\phi^{*}\left(\lambda +\gamma^{\prime }g\left(\theta ,x_{t}\right)\right)\right]\right\}$$

where λ and γ are vectors of Lagrange multipliers and $\phi^{*}$ is the convex conjugate of ϕ (for a convex function f(x), its convex conjugate $f^{*}$ is given by $f^{*}\left(y\right)=\sup_{x}\left[xy-f\left(x\right)\right]$ [38]). After the algebraic development of Equation (11), we obtain an expression that is equivalent to Equation (7), which defines the GEL estimator. Thus, the GEL and GMC estimators share similar properties, such as the same asymptotic distribution, the possibility to use an objective function value for inference, and similar arguments for conducting overidentifying tests [38].

##### Exponentially Tilted Empirical Likelihood (ETEL)

Ref. [47] proposed an estimator that merges the EL estimator, which has good asymptotic properties in the case of correctly specified models, with the ET estimator, which has robust behavior under global misspecification, called exponentially tilted empirical likelihood (ETEL), and that we can define by

$${\hat{\theta }}_{ETEL}=\underset{\theta \in \Theta }{arg min}\left[\frac{1}{T}\sum_{t=1}^{T}\tilde{h}\left({\hat{p}}_{t}\left(\theta \right)\right)\right]$$

where ${\hat{p}}_{t}\left(\theta \right)$ is the solution of

$$\min_{{\left\{p_{t}\right\}}_{t=1}^{T}}\left\{\frac{1}{T}\sum_{t=1}^{T}h\left(p_{t}\right):\sum_{t=1}^{T}p_{t}=1;\;\sum_{t=1}^{T}p_{t}g\left(x_{t},\theta \right)=0\right\}$$

with $\tilde{h}\left(p_{t}\right)=-\ln\left(Tp_{t}\right)$ and $h\left(p_{t}\right)=Tp\ln\left(Tp_{t}\right)$.

This estimator exhibits the same advantages of both estimators that define it; that is, it possesses the same low bias and the same variance of the EL under correct specification and avoids the difficulties related to the EL under global misspecification because it contains in its structure the ET [3,47]. More specifically, ETEL uses ET to obtain ${\hat{p}}_{t}\left(\theta \right)$ and EL to obtain $\hat{\theta }$ [19].

##### Minimum Hellinger Distance Estimator (HD)

According to [53], the sense of seeking a robust estimation for small perturbations is because data may show deviations from the distribution considered for the modeling. The Hellinger distance can be used to measure the divergence between distributions, as elucidated by (8), and it is defined by

$$H\left(P,\mu \right)=\sqrt{\int {\left(p_{\theta }^{\frac{1}{2}}\left(x\right)-p^{\frac{1}{2}}\left(x\right)\right)}^{2}dx},$$

where $P_{\theta }$ and *P* are probability measures with densities $p_{\theta }$ and *p*, respectively.

Ref. [54] discusses the use of estimators based on the minimization of the Hellinger distance for parametric and nonparametric procedures being asymptotically similar to ML and robust to deviations from the correct specification. Ref. [53] obtains an estimator associating the minimization of the Hellinger distance and moment conditions that is computationally convenient and has minimax robustness properties. The minimum Hellinger distance (HD) estimator is semiparametrically efficient and robust in neighborhoods of true $P_{\theta }$ and can be expressed by

$${\hat{\theta }}_{HD}=\underset{\theta \in \Theta }{arg min}H\left(P_{\theta },\hat{P}\right)\;\Rightarrow \;{\hat{\theta }}_{HD}=\underset{\theta \in \Theta }{arg min}\int {\left(p_{\theta }^{\frac{1}{2}}\left(x\right)-{\hat{p}}^{\frac{1}{2}}\left(x\right)\right)}^{2}dx,$$

where $\hat{p}$ is a nonparametric density estimator for *p*, such as a kernel estimator, and $\hat{P}$ is the corresponding estimator for the probability measure of *x*. This estimator is asymptotically equivalent to ML and, thus, efficient if the model’s hypotheses are satisfied. Moreover, if $\gamma =-\frac{1}{2}$ in (10), we obtain the HD estimator [50] and, therefore, it belongs to GEL/GMC families [53].

##### Exponential Tilting Hellinger Distance (ETHD)

Ref. [3] merged the HD estimator, which has good asymptotic properties and good performance under correct specification and local misspecification, with the ET estimator, which has good properties under global misspecification. Thus, they generated the exponential tilting Hellinger distance (ETHD) estimator, which is efficient under correct specification and robust to both local and global misspecification. The ETHD estimator is given by

$${\hat{\theta }}_{ETHD}=\underset{\theta \in \Theta }{arg min}H\left(\hat{p}\left(\theta \right),\hat{p}\right)$$

where $\hat{p}\left(\theta \right)$ is the solution of

$$\min_{{\left\{p_{t}\right\}}_{t=1}^{T}}\left\{\frac{1}{T}\sum_{t=1}^{T}p_{t}\log Tp_{t}:\sum_{t=1}^{T}p_{t}g\left(x_{t},\theta \right)=0;\;\sum_{t=1}^{T}p_{t}=1\right\}$$

Note that the ETHD combines the discrepancy function H(·) of the HD defined in (8) with the implied probabilities of the ET [3].

##### Smoothed Generalized Empirical Likelihood (SGEL) and SEL, SET, SCUE, SETEL, SHD, and SETHD Estimators

Smoothing techniques are integral to enhancing the performance of moment condition-based methods, particularly when dealing with non-smooth or discontinuous moment functions. Such irregularities can cause instability in the objective functions, complicating the optimization process. By applying smoothing, the objective functions become numerically differentiable and exhibit properties akin to convex functions, thereby facilitating more reliable optimization. In scenarios involving discrete variables, such as count data, or when moment conditions are associated with nonparametric functions like kernel regressions, smoothing plays a crucial role. It mitigates abrupt changes or high variability in the empirical likelihood calculations, leading to more stable and accurate estimations. Moreover, smoothing addresses the bias–variance tradeoff inherent in statistical estimations. By averaging over noisy or volatile moment conditions, smoothing reduces the variance of the estimated parameters. However, this process may introduce a slight bias. Careful adjustment of smoothing parameters, such as kernel bandwidth, allows for control over this tradeoff, optimizing the balance between bias and variance. When dealing with non-independent and identically distributed (non-iid) data, smoothing becomes even more critical. Non-iid data often exhibit dependencies or heteroscedasticity, which can violate the assumptions underlying traditional GEL methods. Smoothing techniques help in regularizing these complexities, ensuring that the moment conditions remain applicable and the estimations remain consistent and efficient. For instance, in time-series analysis or panel data models where observations are dependent, smoothing can alleviate issues arising from such dependencies, leading to more robust inference.

The estimators of the smoothed generalized empirical likelihood (SEL) family, that is, GEL estimators with smoothed moment conditions (the distinction between GEL and SGEL is present in [40,55]), depicted in [36,40,55], can deal with time-dependent data problems and heteroscedastic and serially correlated moment conditions replacing $g(x_{t},\theta )$ using a smoothed version of the moment conditions given by

$$g^{\omega }\left(x_{t},\theta \right)=\sum_{j=-m}^{m}\omega \left(j\right)g\left(x_{t-j},\theta \right)$$

where ω(j) is a weighting function normalized for unity, defined from a kernel function, similar to what occurs in the HAC estimators proposed by [34,35], and *m* is a truncation lag that reflects the serial correlation order in $g(x_{t},\theta )$. Using smoothed moment conditions can improve performance in terms of bias even in the case of iid data [40]. Using moment conditions of the form $\sum_{t=1}^{T}p_{t}g^{\omega }\left(x_{t},\theta \right)=0$, we obtain versions with smoothed moments of the EL, ET, CUE, ETEL HD, and ETHD estimators named SEL, SET, SCUE, SETEL, SHD, and SETHD.

##### Specification Tests for GEL/GMC Estimators

While the J test is usually used to verify the GMM specification in over-identified cases, in GEL and GMC estimations, in addition to this test, we can also use the Lagrange multiplier (LM) and likelihood ratio (LR) tests that consider the Lagrange multipliers of Equation (7). J, LM, and LR statistics are asymptotically equivalent. Thus, the LM and LR tests under the null have chi-square asymptotic distribution, as well as the J test [39].

#### 3.1.3. Maximum Likelihood and Bayesian Inference

Likelihood-based methods maximize a likelihood function to obtain the parameters. We rewrite the model in state-space representation to employ this method in DSGE estimation. To guarantee identification, there must be more structural shocks than observable variables. The method will have many optimality properties if the model is correctly specified. For more details about this, see Canova [30]. Since we have a single structural shock in our RBC model, we will use only one observed variable (output $y_{t}$) in the estimation process. In situations like this, it is common to add measurement errors in equations as artificial shocks. However, to maintain the same model in all estimates, we used one observable variable.

Our state-space representation based on the log-linearized solution of the model is given by

(12) $$\begin{array}{c} {\tilde{y}}_{t}=H{\tilde{\xi }}_{t-1} \end{array}$$

(13) $$\begin{array}{c} {\tilde{\xi }}_{t}=F\left(\theta \right){\tilde{\xi }}_{t-1}+G\left(\theta \right)\varepsilon_{t} \end{array}$$

(14) $$\begin{array}{c} \varepsilon_{t}∼N\left(0,\sigma^{2}\right) \end{array}$$

where ${\tilde{y}}_{t}$ denotes a vector of observable variables as deviations from their steady-state values (in our case, only product), ${\tilde{\xi }}_{t}$ is a (1×(m+n)) vector of all variables also as deviations from the steady-state values, with *m* and *s* indicating the number of observable and state variables, respectively, that is, ${\tilde{\xi }}_{t}={\left[{\tilde{y}}_{t}^{\prime },{\tilde{z}}_{t}^{\prime }\right]}^{\prime }$, where ${\tilde{z}}_{t}$ is a vector of state variables (only technological shock).

The matrix *H* in Equation (12) provides the mapping between all variables in state variables and the matrices F(θ) and G(θ), in Equation (13), that contains nonlinear relationships between parameters obtained using the log-linearized solution. As the stochastic process—Equation (14)—follows a normal distribution, we use the Kalman filter to evaluate the likelihood function. In sequence, we estimate the parameters using classical inference, obtaining estimates maximizing the likelihood function, or using Bayesian inference, combining the likelihood function and a prior distribution to obtain the posterior distribution of the parameters.

##### Maximum Likelihood (ML)

The ML estimator of the θ is given by

$${\hat{\theta }}_{ML}=\underset{\theta \in \Theta }{arg max}\ell \left(\theta \right)$$

where ℓ(θ) denotes a log-likelihood function defined by

(15) $$\begin{array}{cc} \ell \left(\theta \right)=\log L\left({\tilde{y}}_{t}|\theta \right)= & -\frac{T}{2}\ln\left(2\pi \right)-\frac{1}{2}\log\left|HP_{t|t-1}H^{\prime }\right|\\ \; & \;-\frac{1}{2}\sum_{t=1}^{T}{\left({\tilde{y}}_{t}-H{\tilde{\xi }}_{t|t-1}\right)}^{\prime }{\left(HP_{t|t-1}H^{\prime }\right)}^{-1}\left({\tilde{y}}_{t}-H{\tilde{\xi }}_{t|t-1}\right) \end{array}$$

where $P_{t|t-1}=E\left({\tilde{\xi }}_{t-1}-{\tilde{\xi }}_{t|t-1}\right){\left({\tilde{\xi }}_{t-1}-{\tilde{\xi }}_{t|t-1}\right)}^{\prime }$ is a variance–covariance matrix. The log-likelihood (15) can be expressed in terms of prediction error decomposition. For more details about DSGE estimation using the ML estimator, see [30].

##### Bayesian Inference (BI)

This method combines the likelihood function and a prior distribution in order to generate a posterior distribution of the parameters using the Bayes rule

$$\pi \left(\theta |{\tilde{y}}_{t}\right)=\frac{L\left({\tilde{y}}_{t}|\theta \right)\pi \left(\theta \right)}{\int L\left({\tilde{y}}_{t}|\theta \right)\pi \left(\theta \right)d\theta }$$

where $\pi (\theta |{\tilde{y}}_{t})$ is the posterior distribution of θ, $L({\tilde{y}}_{t}|\theta )$ is the likelihood function, π(θ) is the prior distribution, and $\int L\left({\tilde{y}}_{t}|\theta \right)\pi \left(\theta \right)d\theta$ is the marginal likelihood [56]. Typical inference objectives, as the mean of the posterior distribution, involve the calculation of $\int g\left(\theta \right)\pi \left(\theta |{\tilde{y}}_{t}\right)d\theta$, where g(θ) represents a function of interest.

To find marginal distributions, we need to solve multiple integrals whose solutions usually require numerical methods. The main method used in the Bayesian estimation of DSGE models is the Markov chain Monte Carlo (MCMC). The MCMC involves constructing a Markov chain in θ that converges into a posterior distribution of interest. The original algorithm used to perform numerical integration using the MCMC was developed by [57] and refined by [58]. The so-called random walk Metropolis–Hastings (RWMH) generates a sequence of estimates that follows a random walk process and defines a posterior density after an initial burn-in. For more detail about the RWMH-MCMC, see [30,31,56,59].

### 3.2. Monte Carlo Design

The RBC model presented in Section 2 has seven structural parameters. Table 1 describes these parameters and their values in simulations. The parameters α and δ were calibrated, that is, in estimation, they were replaced by true values (exact calibration). Thus, we have 5 parameters to estimate: θ={β,γ,b,ρ,σ}. The coefficient *b* was chosen so that the value of the hours worked ratio in the steady state is approximate 13, as in [12]. The coefficient σ was defined to generate endogenous variables at reasonable intervals, as in [7]. The other parameter values are recurrent in the literature.

We defined four data-generating processes (DGPs) to estimate a log-linearized model with normally distributed productivity shock. The four DGPs considered are as follows:

- **DGP I:** The log-linear model in Section 2 with normally distributed productivity shock—Equation (2). Just for this DGP, the estimated model is correctly specified.
- **DGP II:** The log-linear model in Section 2 with productivity shock following Student’s t-distribution with 4 degrees of freedom. This DGP admits extreme events; in this case, the estimated model has a global misspecification once the contamination affects a fixed proportion of the sample.
- **DGP III:** The log-linear model in Section 2 with normally distributed productivity shock contaminated by a single positive outlier of magnitude equal to 5 standard deviations in the middle of the sample (fixed outlier). The estimated model has a local misspecification in this case since the contamination disappears asymptotically.
- **DGP IV:** The log-linear model in Section 2 with normally distributed productivity shock contaminated by multiple outliers. The position of the outliers is drawn from a uniform distribution enabling up to 5% of positive outliers (magnitude equal to 3 standard deviations) and up to 5% of negative outliers (magnitude equal to −3 standard deviations). This is another case in which the estimated model is globally misspecified.

All the procedures were performed using the software R. For simulations of the log-linearized DGPs, we used the package gEcon version 1.0.2. This package uses the method proposed by [29] to obtain the model solution. For all the moment-based estimations, we used the package gmm version 1.6-2, described in [60], and for both the ML and BI estimations, we used the package gEcon_estimation. The results were generated from 2000 sample replications with 200 observations each, corresponding to 50 quarters of data, as performed by [13]. The initialization of all the estimators was performed using the true values of the parameters so that the estimator performances could be compared.

In moment-based estimations, we consider both just- and over-identified cases. The moment conditions used in the estimations are presented in Appendix A. For the just-identified case, we used the first five moment conditions—Equations (A1), (A2), and (A6)–(A8). In the over-identified case, we used all seven moment conditions—the previous equations and Equations (A9) and (A10). The priors used in the Bayesian inference were the following: $\beta ∼\mathrm{Beta}(0.98,0.01^{2})$, $\gamma ∼N(3.2,0.5^{2})$, $b∼N(3.2,1^{2})$, $\rho ∼N(0.9,0.05^{2})$, and $\sigma ∼\mathrm{InvGamma}(0.007,0.005^{2})$. Finally, lower and upper limits were placed in the estimator algorithms (including BI) according to the model’s constraints, that is, 0<β<1, γ>0, b>0, −1<ρ<1, and σ>0.

## 4. Results

In this section, we show tables and figures that summarize the main results of this paper. Table 2 presents the correlations between the moment conditions. Note that some correlations are considerably high even between the original moment conditions—the case of conditions $g_{1}\left(x_{t},\theta \right)$ and $g_{3}\left(x_{t},\theta \right)$, for example. The correlation is greater than 50% between $g_{1}\left(x_{t},\theta \right)$ and $g_{7}\left(x_{t},\theta \right)$ and between $g_{2}\left(x_{t},\theta \right)$ and $g_{6}\left(x_{t},\theta \right)$ in all the DGPs. Despite this, the moment conditions appear to have been informative enough to identify the parameters for most estimators. It should be noted that difficulties arising from using artificial moment conditions are the possibility of stochastic singularity between moment conditions, which could lead to serious implications regarding computational implementation.

In Table 3, we show the results of the J, LM, and LR tests for the validity of the moment conditions of the over-identified moment-based estimators. We observe that while the J test rejected the null “correct specification” at the significance level of 5% between 26.40% and 33.45% for the GMM, the same test rejected almost all the replications for the GEL/GMC estimators. For the latter, the LM and LR tests rejected, at the same level of significance, 63.25% to 69.80% and 75.25% to 79.80% of the specifications, respectively. Therefore, under correct specification, the tests indicated rejection of the null hypothesis. In an experiment not reported here, with the increase in the sample from 200 to 2000 observations, the rejection ratio at 5% of the null in the J test for the GMM dropped to 1.55%, considering the **DGP I**. That is, there was a noticeable performance problem with the test in a small sample. For the tests based on the GEL/GMC estimators, on the other hand, there was no improvement in performance with increasing the sample size.

In econometric analysis, tests such as the likelihood ratio (LR), Lagrange multiplier (LM), and J tests play a central role in evaluating model specification and the validity of moment conditions, particularly in the context of the generalized empirical likelihood (GEL) and generalized method of moments (GMM) estimators. However, these tests often display notable limitations in finite samples, which can lead to an over-rejection of the null hypothesis even when the model is correctly specified.

The J test, widely used to assess the validity of moment conditions, is prone to over-rejecting the null hypothesis under certain conditions. This tendency is often linked to the choice of instruments and the variability inherent in small samples. Over-rejection can lead to misleading conclusions about model misspecification, even when the specified model aligns well with the data. Additionally, in such cases, parameter estimates derived from the GMM may exhibit downward median bias, further complicating statistical inference. Finite-sample studies of the generalized method of moments (GMM) highlight important tradeoffs and challenges. Ref. [61] observed that using short lags for instruments tends to yield nearly asymptotically optimal estimates, while longer lags introduce bias and misleading confidence intervals. He also noted that tests for overidentifying restrictions perform well in small samples but are slightly biased toward accepting the null hypothesis. Ref. [62] found that the J test exhibits minimal size distortion in some cases but is biased toward over-rejection in others, with parameter estimates often showing downward median bias in cases of over-rejection. Ref. [46] emphasized the importance of how moment conditions are weighted, with continuous updating estimators generally showing less bias but sometimes resulting in fat-tailed sample distributions. This affects confidence intervals and the reliability of overidentifying restriction tests.

Similarly, LR and LM tests are sensitive to sample size, with their performance in finite samples often reflecting size distortions. These distortions increase the likelihood of Type I errors, where valid models are incorrectly rejected. Empirical evidence has demonstrated that the sensitivity of these tests to minor deviations in the data or sampling variability is particularly problematic when dealing with small samples, as is often the case in applied econometrics.

The frequent over-rejection of valid models by these tests raises important concerns, as the results may not necessarily indicate genuine model misspecification but rather reflect the limitations of the test methodologies in finite samples. This issue underscores the need for caution in interpreting the outcomes of LR, LM, and J tests, especially in small-sample contexts. To address these challenges, researchers can employ strategies to mitigate the effects of finite-sample limitations. For example, bootstrap methods are often used to generate more accurate critical values that account for the specific characteristics of the sample, reducing the likelihood of over-rejection. Additionally, the careful selection of instruments and the use of alternative testing procedures designed to perform better in small samples can enhance the robustness of inference.

**Figure 1:**
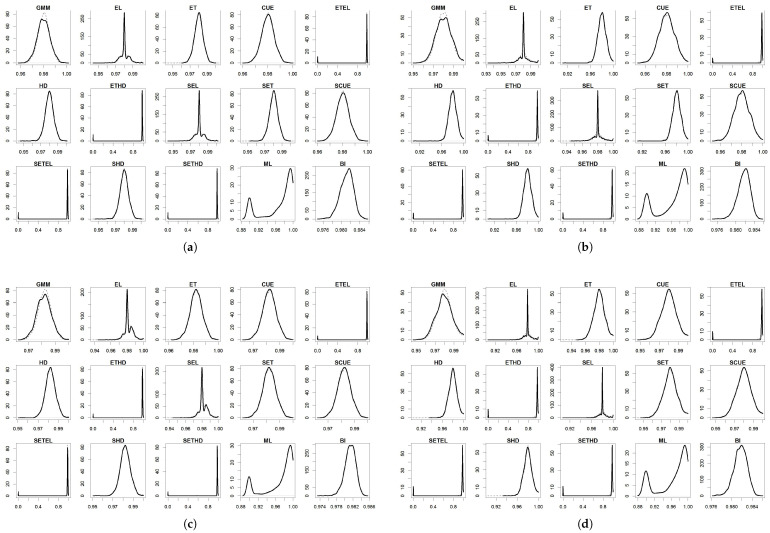
Distribution of $\hat{\beta }$. (**a**) DGP I: normal distribution; (**b**) DGP II: Student’s t-distribution; (**c**) DGP III: normal distribution with single centered outlier; and (**d**) DGP IV: normal distribution with multiple outliers. Notes: True value: β=0.98. Continuous black line: over-identified case. Dashed line: just-identified case.

**Figure 2:**
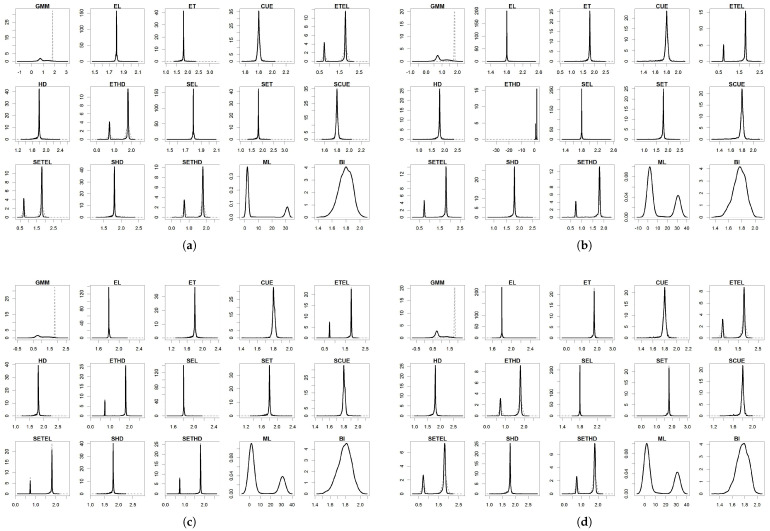
Distribution of $\hat{\gamma }$. (**a**) DGP I: normal distribution; (**b**) DGP II: Student’s t-distribution; (**c**) DGP III: normal distribution with single centered outlier; and (**d**) DGP IV: normal distribution with multiple outliers. Notes: True value: γ=1.8. Continuous black line: over-identified case. Dashed line: just-identified case.

**Figure 3:**
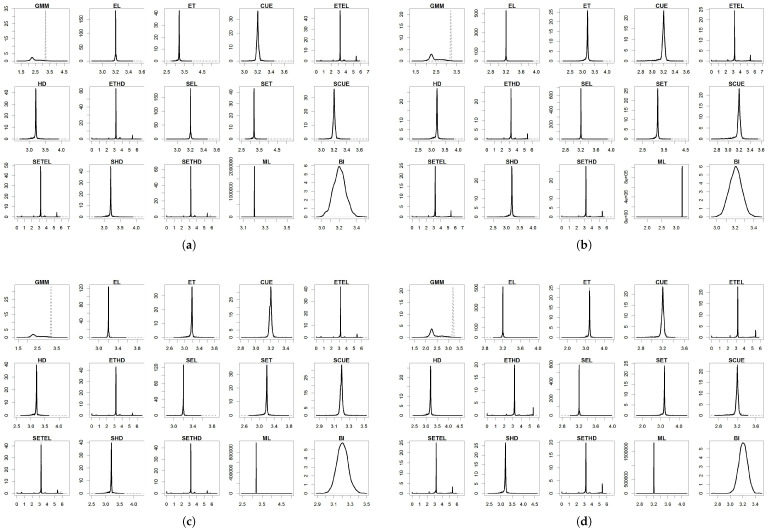
Distribution of $\hat{b}$. (**a**) DGP I: normal distribution; (**b**) DGP II: Student’s t-distribution; (**c**) DGP III: normal distribution with single centered outlier; and (**d**) DGP IV: normal distribution with multiple outliers. Notes: True value: b=3.2. Continuous black line: over-identified case. Dashed line: just-identified case.

**Figure 4:**
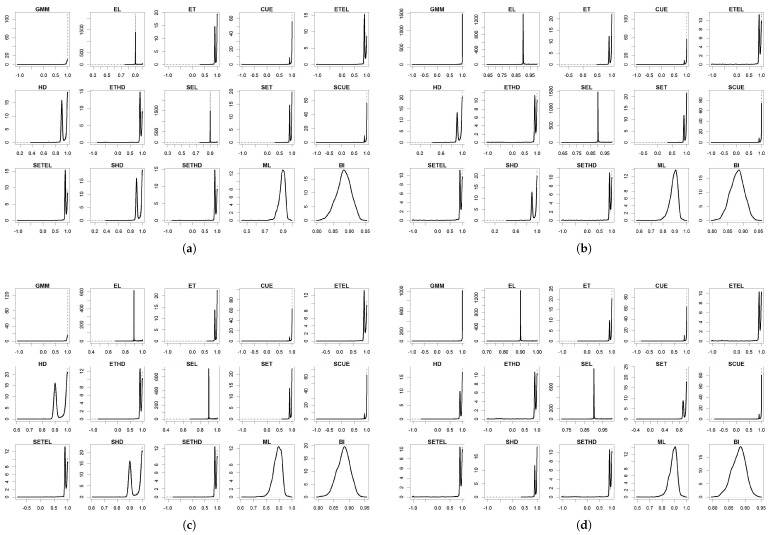
Distribution of $\hat{\rho }$. (**a**) DGP I: normal distribution; (**b**) DGP II: Student’s t-distribution; (**c**) DGP III: normal distribution with single centered outlier; and (**d**) DGP IV: normal distribution with multiple outliers. Notes: True value: ρ=0.9. Continuous black line: over-identified case. Dashed line: just-identified case.

**Figure 5:**
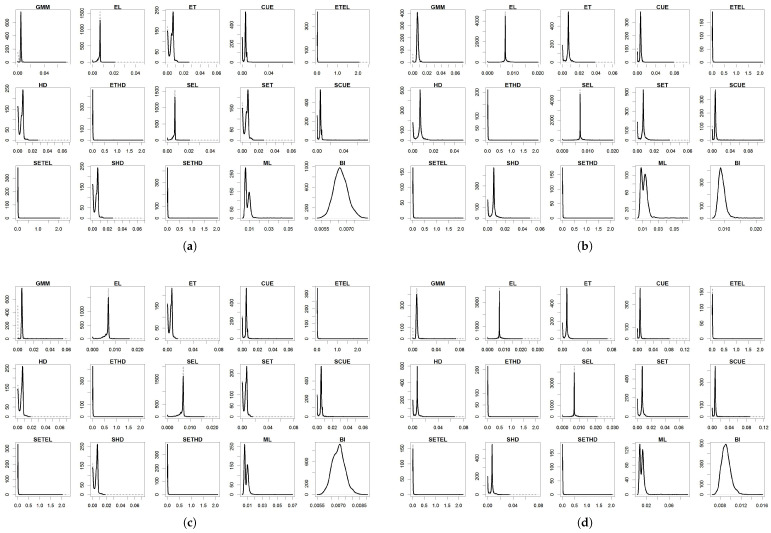
Distribution of $\hat{\sigma }$. (**a**) DGP I: normal distribution; (**b**) DGP II: Student’s t-distribution; (**c**) DGP III: normal distribution single centered outlier; and (**d**) DGP IV: normal distribution with multiple outliers. Notes: True value: σ=0.007. Continuous black line: over-identified case. Dashed line: just-identified case.

For the parameters, the results are summarized in Table 4, Table 5, Table 6, Table 7 and Table 8 and in Figure 1, Figure 2, Figure 3, Figure 4 and Figure 5. To facilitate the description of the results, each parameter appears in one table and one figure, separately distinguishing the DGP considered. In the tables, divided into just- and over-identified cases (for moment-based methods), we have the mean of estimates, median of estimates, bias given by the difference between mean and true parameter, mean squared error (MSE), and mean absolute error (MAE). The figures show the distributions of the parameters generated by each estimator considering 2000 replications. As a matter of space, the distributions generated by both just- and over-identified moment-based methods were superimposed (continuous black and dotted gray lines, respectively). We use the optimization method nlminb to implement the GMM and GEL/GMC estimators. For Bayesian inference (BI), the RWMH-MCMC was constructed with a chain size of 5500 (burn-in of 1500) and maximization routine csminwel. Increases in the size of the chain did not lead to an improvement in results.

The smoothed and non-smoothed versions of the GEL/GMC estimators presented very similar performances, making it impossible to differentiate them. Thus, in several moments, we will mention both versions throughout the text by adding (S) before the estimator’s name. Another general highlight is that the occurrence of extreme estimates for ETEL, ETHD, and their smoothed versions (Figure 1, Figure 2, Figure 3, Figure 4 and Figure 5) deteriorate the mean, MSE, and MAE of these estimators but not the median since this is more robust to the presence of atypical values (Table 4, Table 5, Table 6, Table 7 and Table 8). This behavior was present in the estimation of all the parameters, although it has appeared in different ways: estimates at the limit of the parametric space (parameter β), very discrepant estimates about true values (γ and σ), and both problems (*b* and ρ). The performance problem of these estimators could not be overcome by using other optimization methods available in the gmm package.

The parameters β, γ, and *b* were those for which the estimators analyzed in this paper returned the estimates closer to the true values, except for (i) ETEL, ETHD, and their smoothed versions, for the reason highlighted in the previous paragraph; (ii) GMM that, even considering the median, obtained inferior performance regarding γ and *b* in the over-identified case; and (iii) ML in the case of the γ. In Figure 2 and Figure 3, we see that γ and *b* distributions generated by the over-identified GMM were asymmetric to the right and reasonably spaced, corroborating the poor performance reflected in the statistics contained in Table 5 and Table 6. The GMM’s estimation of the parameters ρ and σ was poor in both the just- and over-identified cases.

In general, the parameters ρ and σ were the most difficult to estimate, and good performance of the (S)EL should be highlighted in obtaining accurate estimates for both. In the case of ρ, a problem with other estimators may derive from the true value (0.9) being close to 1, the upper limit of the parametric space. Much of the σ estimates were biased down, becoming closer to the true value in poor specifications, something expected due to the inclusion of extreme values that lead to increased data variability. Thus, this result should be treated as a coincidence, not a sign of robustness.

Specifically about parameter β, different experiments generated similar results, and the addition of deviations from a normal distribution (of the productivity shock) led to increases in the MSE and MAE of the estimators—most notably in the case of multiple outliers (**DGP IV**) and less in the case of a single outlier (**DGP II**) (Table 4). The lowest MSEs were recorded by BI, GMM, (S)CUE, and (S)EL, while the lowest MAEs were for BI and (S)EL. The problem with (S)ETEL and (S)ETHD was due to the existence of a considerable probability mass close to zero, a lower limit of the parametric space (Figure 1). Even under correct specification (**DGP I**), ML presented poorer performance than the GMM, GEL/GMC, and BI due to the concentration of estimates around 0.9, which may be associated with the existence of a local maximum in the objective function of this estimator.

The estimation of γ is usually the most difficult since this parameter controls the curvature of the utility function, the primary source of nonlinearity in the model, as pointed out by [7,12]. For this parameter, we have that ML and the over-identified GMM presented the worst performance in terms of bias, MSE, and MAE (Table 2). On the other hand, the just-identified GMM presented good performance due to a low MSE and MAE. (S)EL, (S)CUE, and (S)ET delivered the best results in terms of bias, MSE, and MAE, while (S)ETEL and (S)ETHD were among the worst. Estimates of the latter presented concentration around the true value (1.8) and 0.75. Due to a similar problem, ML delivered a greater bias, MSE, and MAE. For ML, estimates were concentrated around 1.8 and 30, a number quite far from the true value.

(S)EL and ML obtained good performance in estimating parameter *b* in all the considered drawings. A clear improvement in the (S)EL was observed from the just-identified to the over-identified case. This improvement did not occur with the (S)CUE, whose performance eventually worsened but not enough for it to leave the list of estimators with the best results (Table 6). The just-identified GMM also generated reasonable estimates compared to the others, especially for data generated by **DGP I**, **DGP III**, and **DGP II**, in this order. Even with an informative prior, the BI delivered wrong estimates for the parameter *b* compared with ML in terms of bias, MSE, and MAE. It occupied an intermediate position in the performance classification of estimators for parameter *b*. The over-identified GMM delivered the best results, while the results of ETEL and (S)ETHD were compromised due to atypical estimates (Figure 3).

The results for ρ presented remarkable peculiarities because almost all the estimators provided estimates concentrated around 0.9 (true value) and 1 (Figure 4). ET, CUE, ETEL, HD, ETHD, and their smoothed versions presented this peculiarity—23 of the analyzed estimators. Despite not having this behavior, the over-identified GMM obtained the largest bias. In contrast, the just-identified GMM obtained one of the largest biases, with the mean and median values close to one in both cases. The main positive performances were registered by EL and SEL, which delivered concentrated estimates only around the true value and the lowest MSE and MAE among all the estimators (Table 7). Despite reporting values close to true, ML generated a reasonably wide distribution around this value. BI, in turn, despite delivering a good MSE, returned more biased estimates than ML, and its MAE was at least twice greater than the MAE of the (S)EL in all the analyzed cases, even counting with an informative prior.

We impose a positivity restriction for σ since this parameter appears squared in the moment conditions, allowing for two solutions: the true 0.007 and the opposite −0.007. Such a feature tends to explain why, in the case of the (S)ET, (S)CUE, (S)HD, (S)ETEL, and (S)ETHD and the GMM (the latter to a lesser extent) reported values close to zero (Figure 5). In a way, the maximization algorithm could have “looked” at the other admitted value (−0.007), and it was prevented from reaching it due to the lower limit imposed. Several GEL/GMC estimators delivered atypical results due to estimates much higher than true in some replications. The ML distribution concentrated around 0.006 and 0.01 in the estimation using data of the **DGP I** and around 0.008 and 0.013 in the case of **DGP IV**. In the correctly specified case (**DGP I**) and the case of contamination by only a sample-centered outlier (**DGP III**), BI delivered one of the best results in terms of bias, MSE, and MAE. It was followed closely by (S)EL (Table 8). In misspecification estimations using **DGP II** and **DGP IV**, the (S)EL estimator tended to outperform BI in terms of bias, MSE, and MAE. Considering the existence of a fixed parameter, (S)EL can be considered more robust than BI in the estimation of σ since it was able to deliver more accurate estimates with a smaller MSE and MAE.

In general, the (S)EL estimator, in both just- and over-identified cases, and BI obtained the best performances in terms of bias, MSE, and MAE, in both correctly specified and misspecified models. Good performance of the EL in a correctly specified model was expected since it has good asymptotic properties in this situation. Because it incorporates prior information, BI generates good results, mainly due to the good configuration of the prior distribution—which was the case since both the prior and initialization of all the estimators were defined from the true values of the parameters. In addition, it is worth noting that, despite the intermediate comparative performance, the performances of the (S)CUE, (S)HD, and (S)ET estimators were shown to be good tools for DSGE estimation.

The GMM, mainly in the over-identified case, and ML presented performances considerably below a good part of their competitors due to inaccurate estimates. The non-zero empirical moment conditions of the over-identified GMM tend to compromise the performance when data are exposed to disturbances. Along this line, the GEL/GMC estimators have the advantage of always satisfying the constraints due to weighting by implicit probability to act directly on observations and not on moment conditions, as in the GMM. The ML estimator delivered concentrated estimates at points other than the true values, which may be associated with the presence of a local maximum in its objective function, showing that despite full information, this method can be reasonably unstable.

We can interpret the inferior results of the GMM by the fact that the generalized empirical likelihood and generalized minimum contrast methods, along with their smoothed variants, offer significant advantages over the generalized method of moments (GMM) estimators. One key advantage is that GEL methods do not require the estimation of a weight matrix, which is essential in the over-identified GMM. Estimating this matrix can be computationally complex and may introduce additional errors, whereas GEL inherently incorporates the moment conditions without relying on such a step, simplifying the process and reducing potential inaccuracies. Smoothed versions of GEL, such as the smoothed empirical likelihood (SEL), further improve estimation by achieving higher-order accuracy in finite samples. Furthermore, GEL methods are nonparametric, making them more flexible and requiring fewer assumptions about the underlying distribution of errors.

The finite-sample properties of the generalized method of moments (GMM) and generalized empirical likelihood (GEL) are further influenced by the methodology used to estimate the GMM parameters, particularly when employing two-step and iterated GMM approaches. These methodologies introduce additional sources of bias and inefficiency, which can significantly impact the reliability of GMM estimators in finite samples.

In the two-step GMM, the estimation process is divided into two stages. The first step involves obtaining an initial estimate of the parameters, often using an identity weighting matrix or some simple approximation. This preliminary estimate is then used to calculate the optimal weighting matrix in the second stage, which minimizes the asymptotic variance of the estimator. While this approach is theoretically efficient asymptotically, in finite samples, the second-stage weighting matrix depends on the first-stage estimates, introducing a form of feedback bias. This bias arises because the variability in the first-stage estimates propagates into the second stage, amplifying the estimation error. The magnitude of this bias grows with the number of moment conditions, as the estimation of the weight matrix becomes less stable when the dimensionality of the moment conditions is highly relative to the sample size.

The iterated GMM aims to mitigate this issue by repeatedly updating the parameter estimates and the weighting matrix until convergence. This iterative procedure improves asymptotic efficiency and reduces the dependence on the initial choice of the weighting matrix. However, in finite samples, the iterated GMM does not fully eliminate the bias introduced by the staged estimation process. The iterated updates can exacerbate finite-sample sensitivity, particularly when the moment conditions are weakly informative or when the sample size is small relative to the number of moments. Furthermore, the iterated GMM can become computationally burdensome, and convergence to a global optimum is not guaranteed in nonlinear settings, adding further practical limitations.

In contrast, generalized empirical likelihood (GEL) methods, such as empirical likelihood (EL), exponential tilting (ET), and continuous updating estimator (CUE), avoid these staged estimation processes entirely. GEL constructs its objective function directly based on the likelihood principle, which ensures that the weighting of the moment conditions is determined endogenously. This approach eliminates the need for pre-estimating a weighting matrix, thereby avoiding the feedback bias inherent in the two-step and iterated GMM. GEL’s continuous updating structure is particularly advantageous in finite samples, as it incorporates all the moment conditions simultaneously without relying on intermediate steps or iterative updates. The CUE, a variant of GEL, further optimizes the empirical likelihood function directly over both the parameters and the moment conditions, offering an efficient and bias-robust alternative to the iterated GMM.

The smoothing techniques applied in GEL also contribute to improved numerical stability, especially in situations where the moment functions are non-smooth or involve discontinuities. This is especially useful in complex models or irregular data structures, where optimization using traditional methods can be challenging. By addressing these issues, GEL and its smoothed variants provide a reliable and efficient framework for estimation, outperforming the GMM in terms of robustness, flexibility, and finite-sample accuracy.

The performance of ET- and HD-based estimators—(S)ETEL and (S)ETHD—was strongly compromised by atypical estimates in some replications. These estimators could deliver good results mainly when comparing the performance of different estimators under specification problems—especially the ETHD estimator that presents good theoretical properties under both correct specification and misspecification. However, it should be emphasized that other studies that consider other sources of misspecification must be made. In addition, other analyses that consider different initialization can be made to analyze the performance of these estimators in other situations. It would be interesting to analyze the choice of optimization methods used in the implementation of estimators, something outside the scope of this paper.

The exponentially tilted empirical likelihood (ETEL) estimator combines the strengths of empirical likelihood (EL) and exponential tilting (ET) to achieve desirable properties under both correct and incorrect model specifications. Ref. [47] demonstrated that ETEL maintains the low bias of EL in correctly specified models while avoiding EL’s issues under misspecification. However, its finite-sample performance can be significantly compromised by atypical estimates caused by extreme values. This behavior often results in estimates clustering at the boundaries of the parametric space or diverging substantially from true values, highlighting its lack of robustness to outliers. Small sample sizes exacerbate this issue, increasing estimator variance and limiting its reliability, which underscores the need for regularization techniques to mitigate these limitations.

Similarly, the exponentially tilted Hellinger distance (ETHD) estimator was developed to address the lack of robustness in traditional Hellinger distance (HD) estimators under global model misspecification. Ref. [3] showed that while HD estimators are efficient under correct specifications, they fail to maintain $\sqrt{n}$-consistency when the model is globally misspecified. ETHD integrates ET to enhance robustness against such misspecifications; however, its finite-sample performance is similarly vulnerable to extreme values, leading to parameter estimates that deviate significantly from the true values. Additionally, ETHD’s sensitivity to tuning parameters and data characteristics, such as contamination and the underlying distribution of estimating functions, further challenges its practical application.

One of the difficulties related to using moment-based methods, as highlighted by [7], is obtaining a sufficient number of moment conditions for estimating DSGE models with many parameters. Resorting to the definition of artificial moment conditions can generate problems in estimation, making it difficult to use those methods. Another limitation of moment-based methods is the impossibility of recovering latent variables as performed in state-space representation used by ML and BI. However, some GEL/GMC estimators can obtain good results even in situations of misspecification, as found in this paper.

Bayesian inference is a powerful tool for estimating dynamic stochastic general equilibrium (DSGE) models, offering a structured approach to incorporate prior knowledge and quantify parameter uncertainty. However, its effectiveness is closely tied to the specification of prior distributions. In situations where the available data are not sufficiently informative, the choice of priors can significantly influence the posterior distributions, potentially leading to results that reflect the imposed priors more than the underlying data. This issue is particularly pronounced in small-sample contexts, where limited data exacerbate the dominance of prior assumptions. Consequently, the reliability of the estimation outcomes may be compromised, as the posterior inferences may be more indicative of the prior configurations than the empirical evidence. Therefore, careful consideration and sensitivity analysis of prior choices are essential to ensure robust and credible parameter estimates in Bayesian estimation of DSGE models.

About BI, despite the advantages of using prior information already mentioned in [13], it should be emphasized that if the data are not informative enough, the prior configuration can largely dominate the posterior distribution. Thus, sensitivity tests involving prior distribution are fundamental to guarantee the robustness of results. In addition, another disadvantage of BI is the time required to obtain the posterior distribution, which is usually considered high.

## 5. Conclusions

The estimation of dynamic stochastic general equilibrium (DSGE) models involves a tradeoff between computational efficiency and the thoroughness of parameter inference. Generalized empirical likelihood (GEL) and generalized method of moments (GMM) estimators are often favored for their computational efficiency. These methods rely on moment conditions derived from the model, allowing for parameter estimation without the need to solve the full likelihood function, which simplifies computations and reduces processing time. However, this efficiency may come at the cost of statistical efficiency, particularly in small samples, where GEL and GMM estimators can exhibit higher variance compared to maximum likelihood (ML) estimators.

In contrast, methods like maximum likelihood (ML) and Bayesian inference (BI) provide a more comprehensive framework for parameter estimation by utilizing the full likelihood function. Bayesian estimation, in particular, offers a systematic approach to incorporate prior information and quantify parameter uncertainty through posterior distributions. This process typically involves Markov chain Monte Carlo (MCMC) methods, which, while powerful, are computationally intensive. The need to sample from complex, high-dimensional posterior distributions can lead to substantial computational times, posing challenges for large-scale models or real-time policy analysis.

To evaluate the performance of different moment-based estimators belonging to generalized empirical likelihood (GEL) and generalized minimum contrast (GMC) families in the estimation of DSGE models, we performed a Monte Carlo analysis considering different data-generating processes to verify the results under both correct and incorrect specifications. As a benchmark, we consider the generalized method of moments (GMM), maximum likelihood (ML), and Bayesian (BI) estimators.

The main results found were the following: (i) the just- and over-identified empirical likelihood (EL) estimator, as well as the smoothed version (SEL), and Bayesian inference (BI), obtained, in this order, the best performances in terms of bias, MSE, and MAE, in situations where the estimated model has and does not have specification problems; (ii) continuous updating empirical likelihood (CUE), the minimum Hellinger distance (HD), exponential tilting (ET), and their smoothed versions presented intermediate comparative performance; (iii) the performance of exponentially tilted empirical likelihood (ETEL), the exponential tilting Hellinger distance (ETHD), and their smoothed versions was strongly compromised by atypical (distant of the true values) estimates in some replications; (iv) smoothed and non-smoothed versions of the GEL/GMC estimators showed very similar performances, so it is impossible to distinguish them; and (v) the GMM estimator, especially in the over-identified case, and the ML estimator presented poor performances due to the inaccurate estimates.

In general, the performance of some GEL/GMC estimators, more specifically EL and its version with smoothed moment conditions, was similar (and in some cases even higher) to the Bayesian estimator and superior to the GMM and ML estimators. We emphasize that EL delivering estimates as good as those of the Bayesian method is an outstanding result because the latter has the advantage of incorporating prior information. However, some difficulties associated with defining the sufficient number of informative moment conditions may make the use of GEL/GMC (and GMM) estimators unfeasible. Since GEL/GMC estimators always satisfy their restrictions, they are considerably more advantageous than the over-identified GMM. On the other hand, Bayesian inference has difficulties such as the prior distribution dominating the final result in small samples and the long time spent in estimation. Thus, GEL/GMC estimators can be good tools for DSGE estimation, given their good characteristics in the misspecification context and their easy and fast computational implementation.

The estimation of parameters using methods such as the generalized method of moments (GMM), continuous updating generalized empirical likelihood (GEL), and generalized minimum contrast estimators (GMC) is often challenged by numerical and optimization instabilities. These challenges primarily arise from the complexity of the optimization landscapes, sensitivity to initial conditions, and the potential presence of multiple local minima. Addressing these issues is critical for enhancing the reliability and robustness of these estimators.

In the GMM, numerical instabilities frequently stem from the estimation of the optimal weighting matrix. When the number of moment conditions is large relative to the sample size, the sample covariance matrix of the moments can become ill-conditioned, leading to unreliable parameter estimates. Two-step and iterated GMM procedures exacerbate these issues, as the feedback loop between the initial parameter estimates and the subsequent weighting matrices amplifies estimation errors. Furthermore, convergence to suboptimal solutions is a recurring problem, particularly in high-dimensional settings or when the moment conditions are weak.

GEL methods, such as the continuous updating estimator (CUE), are designed to address some of the shortcomings of the GMM by jointly estimating parameters and the weighting matrix. However, GEL is not immune to optimization difficulties. These methods often involve solving non-convex optimization problems that are highly sensitive to initial conditions, which increases the risk of converging to local optima. The computational complexity of GEL grows with the dimensionality of the parameter space and the number of moment conditions, further complicating its practical application in large-scale problems.

In the case of GMC, which minimizes a contrast function between the empirical and model-implied distributions, the choice of contrast function plays a crucial role. Poorly chosen contrast functions can lead to optimization surfaces that are flat or irregular, making it difficult for numerical algorithms to converge reliably. The estimation of implied probabilities adds another layer of complexity, as these probabilities often require iterative numerical procedures that can be prone to convergence issues, particularly when starting values are poorly selected.

The accuracy of parameter estimation in these methods depends heavily on the specification of moment conditions and the correct computation of implied probabilities. Misspecified moment conditions result in biased or inconsistent estimators, while errors in computing implied probabilities can further distort parameter estimates. In particular, the iterative procedures used in GEL and GMCE for computing implied probabilities may fail to converge in high-dimensional parameter spaces, complicating the estimation process.

To address these challenges, researchers have proposed various strategies. Regularization techniques, for example, can stabilize the estimation of the weighting matrix in the GMM, especially when dealing with a large number of moment conditions [63]. By incorporating penalty terms into the optimization objective, regularization reduces overfitting and enhances numerical stability. Robust optimization algorithms [64], including quasi-Newton methods, simulated annealing, and adaptive learning rate techniques, are also beneficial for GEL and GMC methods, as they mitigate sensitivity to initial conditions and improve the likelihood of converging to a global optimum. The careful selection of instruments, particularly in the GMM, enhances efficiency and stability by reducing the dimensionality of the problem and mitigating weak identification issues. Additionally, the adoption of advanced numerical methods and high-performance computing strategies, such as parallel processing, can alleviate the computational burden associated with these estimation procedures.

Moment-based estimators can be valuable tools for estimating parameters in dynamic stochastic general equilibrium (DSGE) models due to their flexibility and minimal reliance on distributional assumptions. However, these methods have notable limitations, particularly when dealing with the latent variables that are central to DSGE models.

These estimators rely on aligning theoretical model-implied moments with empirical data moments, but it does not directly estimate latent variables, such as structural shocks or unobservable state variables. This indirect approach can lead to inefficiencies in parameter estimation, especially in cases where latent variables significantly influence the dynamics of the model. Moreover, the inability to explicitly account for latent components introduces challenges in identifying structural parameters, as the available moment conditions may not fully capture the model’s underlying dynamics. This limitation is exacerbated when moment conditions are misspecified, potentially leading to biased or inconsistent parameter estimates.

To address these challenges, moment-based estimators can be augmented with filtering techniques, such as the Kalman filter or particle filters, which are well suited to handling latent variables. For linear DSGE models with Gaussian assumptions, the Kalman filter can be used to estimate the state-space representation, efficiently handling the unobserved components of the model. By combining moment-based methods with the Kalman filter, researchers can iteratively estimate structural parameters while accounting for the latent states, improving the robustness of the estimation.

In the case of nonlinear DSGE models or those involving non-Gaussian features, particle filters provide a flexible alternative. These filters approximate the posterior distribution of latent variables through sequential Monte Carlo methods using the estimated fixed parameters. Combining the GMM, GEL and GMC with particle filters allows for a more comprehensive treatment of latent variables, although this approach requires careful attention to computational demands and convergence properties. By integrating moment-based estimators with filtering techniques, it is possible to overcome the limitations of traditional moment-based estimators in the context of DSGE models. This combined approach enhances the ability to estimate structural parameters accurately while accounting for the latent variables that are integral to the model’s structure, leading to more reliable and efficient results.

## Figures and Tables

**Table 1:** Description and true values of the parameters.

Parameter	Description	Value	Type
α	Output elasticity of the capital	0.33	Calibrated
δ	Capital depreciation rate	0.025	Calibrated
β	Intertemporal discount factor of the utility function	0.98	Estimated
γ	Consumption relative risk aversion	1.8	Estimated
*b*	Weight of leisure in the utility function	3.2	Estimated
ρ	Autoregressive coefficient of the technological process	0.9	Estimated
σ	Standard deviation of the productivity shock	0.007	Estimated

**Table 2:** Correlation between moment conditions.

	$g_{1}$	$g_{2}$	$g_{3}$	$g_{4}$	$g_{5}$	$g_{6}$	$g_{7}$
DGP I: normal distribution							
$g_{1}$	1.0000						
$g_{2}$	−0.1244	1.0000					
$g_{3}$	0.6197	−0.0355	1.0000				
$g_{4}$	0.0346	−0.1543	0.0717	1.0000			
$g_{5}$	−0.0055	−0.0180	0.0205	0.1859	1.0000		
$g_{6}$	−0.2333	0.6505	−0.1311	−0.1252	−0.0166	1.0000	
$g_{7}$	0.5734	−0.2909	0.3468	0.0810	0.0070	−0.3548	1.0000
DGP II: Student’s t-distribution							
$g_{1}$	1.0000						
$g_{2}$	−0.1214	1.0000					
$g_{3}$	0.6062	−0.0351	1.0000				
$g_{4}$	0.0146	−0.2063	0.1296	1.0000			
$g_{5}$	−0.0314	−0.0165	0.0124	0.2505	1.0000		
$g_{6}$	−0.2044	0.6579	−0.1067	−0.1697	−0.0193	1.0000	
$g_{7}$	0.5727	−0.2031	0.3401	0.0321	−0.0375	−0.2268	1.0000
DGP III: normal distribution with single, centered outlier							
$g_{1}$	1.0000						
$g_{2}$	−0.1287	1.0000					
$g_{3}$	0.6178	−0.0422	1.0000				
$g_{4}$	0.1445	−0.1096	0.1725	1.0000			
$g_{5}$	0.1483	−0.0674	0.1159	0.2332	1.0000		
$g_{6}$	−0.2323	0.6572	−0.1365	−0.1119	−0.0923	1.0000	
$g_{7}$	0.5698	−0.2654	0.3527	0.1533	0.3185	−0.3145	1.0000
DGP IV: normal distribution with multiple outliers							
$g_{1}$	1.0000						
$g_{2}$	−0.1136	1.0000					
$g_{3}$	0.6076	−0.0291	1.0000				
$g_{4}$	0.0127	−0.2140	0.0872	1.0000			
$g_{5}$	−0.0389	−0.0080	0.0071	0.2143	1.0000		
$g_{6}$	−0.2153	0.6551	−0.1131	−0.1634	−0.0024	1.0000	
$g_{7}$	0.5700	−0.2477	0.3365	0.0615	−0.0562	−0.2866	1.0000

Notes: The correlation matrix generated from the means of the correlations between the moment conditions generated from the data of each DGP in each replication, considering the true value of the parameters.

**Table 3:** Over-identified J, LM, and LR tests—moment-based methods.

		DGP I	DGP II	DGP III	DGP IV
GMM	*p*-value of the J test (mean)	0.3973	0.4355	0.3948	0.4469
	*p*-value of the J test <0.05	33.45%	26.40%	30.55%	26.85%
GEL/GMC	*p*-value of the J test (mean)	0.0000	0.0000	0.0000	0.0000
	*p*-value of the J test <0.05	99.35%	99.95%	99.55%	99.75%
	*p*-value of the LM test (mean)	0.1100	0.0905	0.1131	0.1108
	*p*-value of the LM test <0.05	64.55%	69.80%	63.25%	66.85%
	*p*-value of the LR test (mean)	0.0603	0.0589	0.0609	0.0718
	*p*-value of the LR test <0.05	79.80%	77.30%	78.20%	75.25%

Notes: Null hypothesis of J, LM, and LR tests: correct specification (moment conditions are valid).

**Table 4:** Statistics of $\hat{\beta }$.

	$\hat{\beta }$	GMM	EL	ET	CUE	ETEL	HD	ETHD	SEL	SET	SCUE	SETEL	SHD	SETHD	ML	BI
		Just-Identified Case														
DGP I	Mean	0.980202	0.980078	0.980189	0.980193	0.948072	0.980139	0.949710	0.980113	0.980186	0.980201	0.947588	0.980171	0.948761	0.969253	0.981141
	Median	0.980256	0.980000	0.980210	0.980211	0.980161	0.980173	0.980162	0.980000	0.980210	0.980204	0.980176	0.980173	0.980186	0.985471	0.981258
	Bias	0.000202	**0.000078**	0.000189	0.000193	−0.031928	0.000139	−0.030290	0.000113	0.000186	0.000201	−0.032412	0.000171	−0.031239	−0.010747	0.001141
	MSE	0.000025	0.000032	0.000026	0.000025	0.031716	0.000028	0.029810	0.000031	0.000026	0.000025	0.032200	0.000028	0.030771	0.001354	**0.000003**
	MAE	0.003931	0.003603	0.003966	0.003971	0.036061	0.003996	0.034450	0.003514	0.003964	0.003967	0.036545	0.003980	0.035406	0.025420	**0.001502**
DGP II	Mean	0.980317	0.980663	0.980307	0.980314	0.950247	0.980324	0.948073	0.980701	0.980340	0.980315	0.946758	0.980360	0.944806	0.963870	0.981688
	Median	0.980272	0.980000	0.980229	0.980299	0.980245	0.980228	0.980174	0.980000	0.980210	0.980319	0.980164	0.980266	0.980219	0.979883	0.981800
	Bias	0.000317	0.000663	0.000307	0.000314	−0.029753	0.000324	−0.031927	0.000701	0.000340	0.000315	−0.033242	0.000360	−0.035194	−0.016130	0.001688
	MSE	0.000051	0.000056	0.000052	0.000051	0.02863	0.000054	0.029954	0.000052	0.000051	0.000052	0.032034	0.000052	0.033277	0.001581	**0.000004**
	MAE	0.005673	0.005058	0.005693	0.005744	0.035698	0.005714	0.037844	0.004733	0.005644	0.005750	0.039032	0.005661	0.041033	0.027738	**0.001845**
DGP III	Mean	0.982165	0.981722	0.982152	0.982186	0.951919	0.982170	0.952232	0.981664	0.982168	0.982187	0.951929	0.982182	0.952254	0.970715	0.981311
	Median	0.982222	0.980124	0.982183	0.982235	0.982044	0.982150	0.982072	0.980067	0.982201	0.982235	0.982016	0.982177	0.982067	0.985982	0.981383
	Bias	0.002165	0.001722	0.002152	0.002186	−0.028081	0.002170	−0.027768	0.001664	0.002168	0.002187	−0.028071	0.002182	−0.027746	−0.009285	**0.001311**
	MSE	0.000030	0.000034	0.000030	0.000030	0.029804	0.000032	0.029292	0.000033	0.000030	0.000030	0.029804	0.000032	0.029291	0.001227	**0.000003**
	MAE	0.004334	0.003932	0.004349	0.004393	0.034583	0.004391	0.034297	0.003813	0.004346	0.004389	0.034573	0.004392	0.034260	0.024026	**0.001565**
DGP IV	Mean	0.979943	0.980186	0.97992	0.979946	0.940299	0.979952	0.940896	0.980216	0.979970	0.979951	0.934493	0.979966	0.934347	0.964714	0.981702
	Median	0.979914	0.980000	0.979961	0.979980	0.980000	0.980000	0.980000	0.980000	0.979975	0.979980	0.980000	0.980002	0.979998	0.980867	0.981757
	Bias	−0.000057	0.000186	−0.000080	−0.000054	−0.039701	−0.000048	−0.039104	0.000216	**−0.000030**	−0.000049	−0.045507	−0.000034	−0.045653	−0.015286	0.001702
	MSE	0.000062	0.000068	0.000064	0.000063	0.037253	0.000066	0.035875	0.000064	0.000062	0.000063	0.043270	0.000067	0.042567	0.001558	**0.000004**
	MAE	0.006210	0.005552	0.006241	0.006286	0.045813	0.006164	0.045107	0.005208	0.006173	0.006271	0.051552	0.006173	0.051570	0.027472	**0.001832**
		Over-identified Case														
DGP I	Mean	0.980038	0.980161	0.980218	0.980197	0.944520	0.980174	0.945539	0.980157	0.980226	0.980194	0.943096	0.980195	0.944580	–	–
	Median	0.979968	0.980000	0.980292	0.980261	0.980219	0.980211	0.980182	0.980000	0.980293	0.980260	0.980231	0.980215	0.980202	–	–
	Bias	**0.000038**	0.000161	0.000218	0.000197	−0.035480	0.000174	−0.034461	0.000157	0.000226	0.000194	−0.036904	0.000195	−0.035420	–	–
	MSE	0.000029	0.000026	0.000025	0.000025	0.035297	0.000026	0.034340	0.000026	0.000025	0.000025	0.036730	0.000025	0.035307	–	–
	MAE	0.004316	0.003291	0.003911	0.003963	0.039577	0.003925	0.038645	0.003274	0.003904	0.003963	0.041014	0.003906	0.039606	–	–
DGP II	Mean	0.980187	0.980143	0.980303	0.980307	0.953273	0.980288	0.950850	0.980192	0.980312	0.980276	0.946421	0.980293	0.945478	–	–
	Median	0.980179	0.980000	0.980298	0.980197	0.980215	0.980239	0.980209	0.980000	0.980218	0.980230	0.980203	0.980204	0.980209	–	–
	Bias	0.000187	**0.000143**	0.000303	0.000307	−0.026727	0.000288	−0.02915	0.000192	0.000312	0.000276	−0.033579	0.000293	−0.034522	–	–
	MSE	0.000058	0.000048	0.000052	0.000050	0.026950	0.000055	0.029351	0.000043	0.000052	0.000050	0.033630	0.000053	0.034634	–	–
	MAE	0.006142	0.004083	0.005652	0.005625	0.032777	0.005698	0.035260	0.003846	0.005616	0.005662	0.039508	0.005637	0.040545	–	–
DGP III	Mean	0.981652	0.981628	0.982162	0.982186	0.960641	0.982159	0.960445	0.981600	0.982146	0.982192	0.958674	0.982148	0.959024	–	–
	Median	0.981750	0.980052	0.982144	0.982225	0.982045	0.982106	0.982121	0.980038	0.982095	0.982226	0.982023	0.982088	0.982086	–	–
	Bias	0.001652	0.001628	0.002162	0.002186	−0.019359	0.002159	−0.019555	0.001600	0.002146	0.002192	−0.021326	0.002148	−0.020976	–	–
	MSE	0.000032	0.000032	0.000030	0.000030	0.021252	0.000031	0.021338	0.000031	0.000029	0.000030	0.023181	0.000031	0.022781	–	–
	MAE	0.004499	0.003670	0.004319	0.004357	0.025802	0.004326	0.026085	0.003625	0.004305	0.004364	0.027727	0.004320	0.027511	–	–
DGP IV	Mean	0.979724	0.980046	0.979963	0.979913	0.940565	0.979977	0.941524	0.980121	0.979986	0.979909	0.936183	0.980000	0.936240	–	–
	Median	0.979470	0.980000	0.979926	0.979982	0.979991	0.980000	0.979978	0.980000	0.979973	0.979943	0.979997	0.980000	0.979997	–	–
	Bias	−0.000276	0.000046	−0.000037	−0.000087	−0.039435	−0.000023	−0.038476	0.000121	−0.000014	−0.000091	−0.043817	**0.000000**	−0.043760	–	–
	MSE	0.000072	0.000053	0.000063	0.000063	0.039045	0.000062	0.038090	0.000051	0.000062	0.000063	0.043397	0.000062	0.043382	–	–
	MAE	0.006778	0.004224	0.006206	0.006229	0.045453	0.006120	0.044554	0.004069	0.006133	0.006259	0.049747	0.006091	0.049777	–	–

Notes: True value: β=0.98. MSE = mean squared error. MAE = mean absolute error. Best performances highlighted in bold.

**Table 5:** Statistics of $\hat{\gamma }$.

	$\hat{\gamma }$	GMM	EL	ET	CUE	ETEL	HD	ETHD	SEL	SET	SCUE	SETEL	SHD	SETHD	ML	BI
		Just-Identified Case														
DGP I	Mean	1.799693	1.799975	1.792883	1.801694	1.517973	1.789484	1.520552	1.799885	1.792690	1.801730	1.517999	1.788053	1.521383	8.831374	1.792836
	Median	1.799847	1.800000	1.798517	1.800003	1.797567	1.798218	1.797667	1.800000	1.798568	1.800000	1.797610	1.798259	1.797722	1.800939	1.798497
	Bias	−0.000307	**−0.000025**	−0.007117	0.001694	−0.282027	−0.010516	−0.279448	−0.000115	−0.007310	0.001730	−0.282001	−0.011947	−0.278617	7.031374	−0.007164
	MSE	**0.000159**	0.000457	0.005423	0.000744	0.302007	0.005682	0.297097	0.000390	0.005747	0.000827	0.304036	0.005929	0.296932	201.512589	0.008871
	MAE	0.009969	**0.008081**	0.030347	0.015673	0.296491	0.032892	0.291800	**0.007773**	0.030736	0.015950	0.298052	0.033481	0.291862	7.037228	0.075397
DGP II	Mean	1.790962	1.802843	1.784633	1.799995	1.562523	1.787709	1.543238	1.802797	1.785611	1.799233	1.557556	1.786721	1.557903	11.134495	1.765028
	Median	1.798876	1.800000	1.796364	1.799511	1.795352	1.796417	1.796424	1.800000	1.796654	1.799148	1.795845	1.796586	1.796864	1.802347	1.771187
	Bias	−0.009038	0.002843	−0.015367	**−0.000005**	−0.237477	−0.012291	−0.256762	0.002797	−0.014389	−0.000767	−0.242444	−0.013279	−0.242097	9.334495	−0.034972
	MSE	0.009343	0.002608	0.007473	0.001485	0.252253	0.008455	1.003001	0.002351	0.007396	**0.002006**	0.256492	0.009235	0.256017	267.848853	0.010563
	MAE	0.022471	**0.014061**	0.040824	0.021177	0.258409	0.041654	0.276881	**0.013076**	0.042633	0.024315	0.262812	0.046638	0.263166	9.343180	0.080309
DGP III	Mean	1.804236	1.803202	1.797763	1.805942	1.617643	1.795489	1.615771	1.803007	1.797153	1.805796	1.617781	1.795471	1.616305	9.788699	1.791399
	Median	1.804486	1.800012	1.80042	1.803724	1.799930	1.800569	1.799717	1.800010	1.800323	1.803240	1.799989	1.800384	1.799834	1.801151	1.797475
	Bias	0.004236	0.003202	**−0.002237**	0.005942	−0.182357	−0.004511	−0.184229	0.003007	−0.002847	0.005796	−0.182219	−0.004529	−0.183695	7.988699	−0.008601
	MSE	**0.000193**	0.000811	0.005644	0.000598	0.196766	0.006879	0.196158	0.000833	0.005667	0.000689	0.196022	0.007093	0.196446	228.820304	0.008040
	MAE	0.010722	**0.009778**	0.034589	0.015518	0.203114	0.036877	0.203922	**0.009648**	0.034742	0.016012	0.202060	0.037444	0.204179	7.991051	0.071078
DGP IV	Mean	1.789161	1.800556	1.787464	1.801799	1.516480	1.786322	1.516122	1.800591	1.786802	1.801877	1.519030	1.782599	1.515419	11.041091	1.765867
	Median	1.798031	1.800000	1.796905	1.799235	1.795199	1.796815	1.79529	1.800000	1.796737	1.799215	1.795556	1.796533	1.795410	1.802214	1.770485
	Bias	−0.010839	**0.000556**	−0.012536	0.001799	−0.283520	−0.013678	−0.283878	**0.000591**	−0.013198	0.001877	−0.280970	−0.017401	−0.284581	9.241091	−0.034133
	MSE	0.012034	**0.001221**	0.006067	0.001660	0.300445	0.007903	0.300181	0.001583	0.006921	0.002633	0.298337	0.008742	0.299022	264.771045	0.009900
	MAE	0.024569	**0.013409**	0.040151	0.023620	0.303472	0.042948	0.304317	**0.013033**	0.042679	0.027369	0.301683	0.046423	0.303688	9.243935	0.078666
		Over-identified Case														
DGP I	Mean	0.975127	1.799331	1.790890	1.799096	1.545488	1.791174	1.543650	1.799398	1.790358	1.799081	1.544086	1.792024	1.540940	–	–
	Median	0.802189	1.800000	1.798327	1.799436	1.797238	1.798465	1.797255	1.800000	1.798364	1.799348	1.797353	1.798663	1.797329	–	–
	Bias	−0.824873	**−0.000669**	−0.009110	−0.000904	−0.254512	−0.008826	−0.256350	**−0.000602**	−0.009642	−0.000919	−0.255914	−0.007976	−0.259060	–	–
	MSE	0.846990	**0.000386**	0.003447	0.000529	0.268875	0.004006	0.273178	**0.000380**	0.003687	0.000545	0.270587	0.004033	0.276147	–	–
	MAE	0.838655	**0.007503**	0.026360	0.013817	0.264018	0.029932	0.264879	**0.007348**	0.026980	0.014069	0.265488	0.030121	0.267831	–	–
DGP II	Mean	0.942684	1.800607	1.785142	1.787255	1.597989	1.785453	1.598327	1.800606	1.784015	1.785070	1.589154	1.783984	1.587769	–	–
	Median	0.760290	1.800000	1.796400	1.797115	1.795194	1.796195	1.795475	1.800000	1.796418	1.796708	1.795443	1.796340	1.795643	–	–
	Bias	−0.857316	**0.000607**	−0.014858	−0.012745	−0.202011	−0.014547	−0.201673	**0.000606**	−0.015985	−0.014930	−0.210846	−0.016016	−0.212231	–	–
	MSE	0.862640	**0.001114**	0.005384	0.003326	0.211625	0.005592	0.208465	**0.001104**	0.005685	0.003911	0.219813	0.006165	0.219402	–	–
	MAE	0.860122	**0.009364**	0.034491	0.027901	0.219019	0.035744	0.215103	**0.008969**	0.036143	0.030250	0.227426	0.036803	0.226450	–	–
DGP III	Mean	1.025254	1.803290	1.794459	1.803422	1.614435	1.794884	1.614503	1.803445	1.794349	1.803941	1.609770	1.794074	1.609564	–	–
	Median	0.901899	1.800022	1.800329	1.802420	1.799357	1.800497	1.799078	1.800019	1.800276	1.802420	1.799118	1.800410	1.799221	–	–
	Bias	−0.774746	**0.003290**	−0.005541	**0.003422**	−0.185565	−0.005116	−0.185497	**0.003445**	−0.005651	**0.003941**	−0.190230	−0.005926	−0.190436	–	–
	MSE	0.760407	**0.000518**	0.003611	0.000688	0.194829	0.003873	0.194965	**0.000500**	0.003689	0.000718	0.199175	0.003993	0.200495	–	–
	MAE	0.782170	**0.008422**	0.028404	0.014609	0.199120	0.029348	0.199320	**0.008289**	0.028818	0.014991	0.202940	0.029664	0.204362	–	–
DGP IV	Mean	0.898878	1.799869	1.781688	1.788705	1.546246	1.785396	1.551558	1.800094	1.781010	1.785889	1.538394	1.782554	1.542250	–	–
	Median	0.745955	1.800000	1.796789	1.797067	1.793437	1.796818	1.794452	1.800000	1.796731	1.797058	1.794026	1.796687	1.794737	–	–
	Bias	−0.901122	**−0.000131**	−0.018312	−0.011295	−0.253754	−0.014604	−0.248442	**0.000094**	−0.018990	−0.014111	−0.261606	−0.017446	−0.257750	–	–
	MSE	0.929671	**0.000673**	0.009571	0.002538	0.260248	0.007289	0.258372	**0.000634**	0.009999	0.003348	0.269271	0.008165	0.269135	–	–
	MAE	0.902122	**0.009113**	0.039962	0.026311	0.264210	0.037998	0.260744	**0.008555**	0.041524	0.029143	0.272321	0.041261	0.270135	–	–

Notes: True value: γ=1.8. MSE = mean squared error. MAE = mean absolute error. Best performances highlighted in bold.

**Table 6:** Statistics of $\hat{b}$.

	$\hat{b}$	GMM	EL	ET	CUE	ETEL	HD	ETHD	SEL	SET	SCUE	SETEL	SHD	SETHD	ML	BI
		Just-Identified Case														
DGP I	Mean	3.199884	3.199865	3.193806	3.202063	3.413509	3.190354	3.462055	3.199669	3.193654	3.202062	3.414318	3.188867	3.461894	3.200015	3.202415
	Median	3.200125	3.200000	3.199589	3.200475	3.200059	3.199401	3.200000	3.200000	3.199560	3.200475	3.200100	3.199290	3.199999	3.200000	3.201663
	Bias	−0.000116	−0.000135	−0.006194	0.002063	0.213509	−0.009646	0.262055	−0.000331	−0.006346	0.002062	0.214318	−0.011133	0.261894	**0.000015**	0.002415
	MSE	0.000146	0.000506	0.006782	0.000740	0.828345	0.006190	1.072016	0.000450	0.007245	0.000795	0.833280	0.006363	1.072546	**0.000077**	0.004821
	MAE	0.009543	0.007671	0.031598	0.015050	0.419455	0.033241	0.493976	0.007374	0.032265	0.015234	0.421944	0.033766	0.493878	**0.000675**	0.054850
DGP II	Mean	3.193172	3.202944	3.186207	3.201210	3.465549	3.189102	3.462018	3.202640	3.187141	3.200331	3.464307	3.188125	3.463299	3.199069	3.202298
	Median	3.199940	3.200000	3.199230	3.200403	3.200053	3.199283	3.200083	3.200000	3.199033	3.200015	3.200027	3.199096	3.200060	3.200001	3.202706
	Bias	−0.006828	0.002944	−0.013793	0.001210	0.265549	−0.010898	0.262018	0.002640	−0.012859	**0.000331**	0.264307	−0.011875	0.263299	−0.000931	0.002298
	MSE	0.005595	0.002546	0.007847	**0.001503**	0.697829	0.008611	0.681449	0.002331	0.008440	0.001915	0.730616	0.009693	0.708161	**0.001380**	0.004512
	MAE	0.020851	0.013128	0.041392	0.020983	0.389054	0.041827	0.385872	0.012139	0.043785	0.023493	0.399738	0.047224	0.396107	**0.001667**	0.053449
DGP III	Mean	3.195213	3.197159	3.18985	3.197132	3.276241	3.187668	3.280302	3.197210	3.189151	3.196983	3.276958	3.187688	3.280464	3.200411	3.205173
	Median	3.195590	3.199981	3.197738	3.197364	3.197940	3.197496	3.197652	3.199990	3.197682	3.197467	3.197975	3.197585	3.197748	3.200000	3.204919
	Bias	−0.004787	−0.002841	−0.010150	−0.002868	0.076241	−0.012332	0.080302	−0.002790	−0.010849	−0.003017	0.076958	−0.012312	0.080464	**0.000411**	0.005173
	MSE	**0.000189**	0.000788	0.006016	0.000523	0.650395	0.007434	0.765980	0.000820	0.006031	0.000605	0.659612	0.007678	0.775130	0.002022	0.004634
	MAE	0.010496	0.008899	0.035644	0.014690	0.319243	0.038253	0.351515	0.008853	0.035874	0.015123	0.321441	0.039104	0.353929	**0.002041**	0.053944
DGP IV	Mean	3.194011	3.201326	3.190618	3.204899	3.463513	3.189162	3.469584	3.201103	3.189636	3.205004	3.461549	3.185192	3.466104	3.200931	3.204142
	Median	3.200960	3.200000	3.199360	3.201902	3.200007	3.199550	3.200118	3.200000	3.199380	3.201667	3.200000	3.199016	3.200034	3.200001	3.204788
	Bias	−0.005989	0.001326	−0.009382	0.004899	0.263513	−0.010838	0.269584	0.001103	−0.010364	0.005004	0.261549	−0.014808	0.266104	**0.000931**	0.004142
	MSE	0.007760	0.001182	0.006176	0.001472	0.813066	0.007779	0.827678	0.001618	0.006836	0.002460	0.828457	0.008719	0.844462	**0.000950**	0.004668
	MAE	0.022339	0.012600	0.038946	0.022482	0.452491	0.041931	0.463055	0.012175	0.041362	0.026344	0.454318	0.045508	0.466301	**0.001952**	0.054291
		Over-identified Case														
DGP I	Mean	2.464474	3.199268	3.191114	3.199251	3.314573	3.191413	3.272090	3.199254	3.190531	3.199261	3.314343	3.192280	3.271027	–	–
	Median	2.311940	3.200000	3.199466	3.199917	3.199976	3.199405	3.199872	3.200000	3.199416	3.199969	3.199949	3.199415	3.199850	–	–
	Bias	−0.735526	−0.000732	−0.008886	−0.000749	0.114573	−0.008587	0.072090	−0.000746	−0.009469	−0.000739	0.114343	−0.007720	0.071027	–	–
	MSE	0.656621	0.000392	0.003665	0.000532	0.659170	0.004101	0.620576	0.000388	0.003899	0.000543	0.667537	0.004193	0.627244	–	–
	MAE	0.749113	0.007005	0.026886	0.013665	0.350254	0.029572	0.327359	0.006917	0.027364	0.013814	0.353666	0.029908	0.330982	–	–
DGP II	Mean	2.433100	3.200452	3.186176	3.188174	3.393048	3.186660	3.347449	3.200266	3.185303	3.185941	3.398548	3.185294	3.347984	–	–
	Median	2.279735	3.200000	3.198486	3.198995	3.199612	3.198932	3.199654	3.200000	3.198605	3.198209	3.199465	3.198911	3.199654	–	–
	Bias	−0.766900	0.000452	−0.013824	−0.011826	0.193048	−0.013340	0.147449	**0.000266**	−0.014697	−0.014059	0.198548	−0.014706	0.147984	–	–
	MSE	0.673377	**0.001020**	0.004908	0.003183	0.654895	0.005498	0.555907	**0.001005**	0.005434	0.003705	0.681973	0.006158	0.583170	–	–
	MAE	0.769747	0.008557	0.033594	0.027396	0.346905	0.034744	0.304845	0.008126	0.035366	0.029322	0.359956	0.035966	0.318940	–	–
DGP III	Mean	2.502882	3.197154	3.18606	3.194563	3.215490	3.186630	3.183700	3.197525	3.186041	3.195114	3.219098	3.185896	3.191682	–	–
	Median	2.387652	3.199984	3.197326	3.196381	3.197470	3.197393	3.197174	3.199985	3.197486	3.196587	3.197487	3.197393	3.197343	–	–
	Bias	−0.697118	−0.002846	−0.013940	−0.005437	0.015490	−0.013370	−0.016300	−0.002475	−0.013959	−0.004886	0.019098	−0.014104	−0.008318	–	–
	MSE	0.594862	**0.000472**	0.003889	0.000701	0.611752	0.004217	0.595892	**0.000429**	0.003979	0.000756	0.614552	0.004335	0.602757	–	–
	MAE	0.703704	0.007846	0.029354	0.014481	0.304345	0.030416	0.287495	0.007619	0.029846	0.014909	0.307208	0.030780	0.291867	–	–
DGP IV	Mean	2.397272	3.200627	3.184836	3.191540	3.395873	3.188472	3.369161	3.200856	3.184097	3.188642	3.402470	3.185503	3.365204	–	–
	Median	2.269591	3.200000	3.198878	3.199785	3.199529	3.199172	3.199620	3.200000	3.199077	3.199663	3.199588	3.199040	3.199674	–	–
	Bias	−0.802728	**0.000627**	−0.015164	−0.008460	0.195873	−0.011528	0.169161	**0.000856**	−0.015903	−0.011358	0.202470	−0.014497	0.165204	–	–
	MSE	0.720260	**0.000677**	0.007963	0.002452	0.873043	0.007532	0.794660	**0.000655**	0.008475	0.003186	0.873846	0.008382	0.794879	–	–
	MAE	0.803583	0.008563	0.038011	0.025349	0.440137	0.036970	0.408439	0.008090	0.039594	0.028180	0.444492	0.040060	0.413237	–	–

Notes: True value: b=3.2. MSE = mean squared error. MAE = mean absolute error. Best performances highlighted in bold.

**Table 7:** Statistics of $\hat{\rho }$.

	$\hat{\rho }$	GMM	EL	ET	CUE	ETEL	HD	ETHD	SEL	SET	SCUE	SETEL	SHD	SETHD	ML	BI
		Just-Identified Case														
DGP I	Mean	0.977691	0.905463	0.962346	0.970118	0.930628	0.955653	0.911981	0.905313	0.962104	0.970083	0.929392	0.954724	0.909244	0.885215	0.882414
	Median	1.000000	0.900010	0.994497	0.999792	0.903631	0.984479	0.932966	0.900008	0.994402	0.999711	0.904164	0.983456	0.931873	0.890047	0.883143
	Bias	0.077691	0.005463	0.062346	0.070118	0.030628	0.055653	0.011981	**0.005313**	0.062104	0.070083	0.029392	0.054724	0.009244	−0.014785	−0.017586
	MSE	0.022264	0.000752	0.008883	0.016600	0.010309	0.005672	0.044984	**0.000732**	0.008707	0.016132	0.012004	0.005912	0.049510	0.001486	**0.000795**
	MAE	0.100184	0.008380	0.068069	0.090834	0.049667	0.057399	0.078945	**0.008138**	0.067771	0.089397	0.050531	0.057942	0.080793	0.027179	0.022399
DGP II	Mean	0.983091	0.907387	0.967626	0.979253	0.891216	0.964043	0.842339	0.906715	0.967469	0.979118	0.895209	0.964862	0.846937	0.889877	0.880486
	Median	1.000000	0.900043	0.996573	1.000000	0.957732	0.993501	0.969795	0.900025	0.996124	1.000000	0.955016	0.994403	0.961090	0.894430	0.881699
	Bias	0.083091	**0.007387**	0.067626	0.079253	−0.008784	0.064043	−0.057661	**0.006715**	0.067469	0.079118	−0.004791	0.064862	−0.053063	−0.010123	−0.019514
	MSE	0.014973	**0.000569**	0.008469	0.013388	0.085466	0.006586	0.167418	**0.000547**	0.008481	0.012713	0.081810	0.006852	0.162462	0.001148	0.000867
	MAE	0.096762	**0.009289**	0.072392	0.094864	0.109091	0.066234	0.161276	**0.008889**	0.072837	0.094106	0.103914	0.068112	0.154297	0.025078	0.023813
DGP III	Mean	0.983464	0.908279	0.966591	0.973547	0.937479	0.961133	0.928809	0.907856	0.966431	0.973083	0.935635	0.961078	0.929809	0.886046	0.881534
	Median	1.000000	0.900023	0.995427	0.999768	0.941139	0.991040	0.967216	0.900018	0.995646	0.999730	0.936131	0.990763	0.963602	0.889886	0.882761
	Bias	0.083464	**0.008279**	0.066591	0.073547	0.037479	0.061133	0.028809	**0.007856**	0.066431	0.073083	0.035635	0.061078	0.029809	−0.013954	−0.018466
	MSE	0.013369	**0.000791**	0.009222	0.014312	0.011114	0.005897	0.037216	**0.000762**	0.009285	0.014579	0.011180	0.005861	0.033622	0.001299	0.000786
	MAE	0.095871	**0.010349**	0.071642	0.091117	0.056678	0.062294	0.075437	**0.009907**	0.072016	0.090623	0.056398	0.062083	0.073001	0.026841	0.022367
DGP IV	Mean	0.979796	0.909221	0.968372	0.974015	0.854999	0.963270	0.813782	0.908104	0.968334	0.975644	0.868764	0.962662	0.826830	0.888880	0.880070
	Median	1.000000	0.900084	0.996954	1.000000	0.943716	0.993773	0.954769	0.900052	0.996397	1.000000	0.938282	0.993448	0.951269	0.893572	0.881925
	Bias	0.079796	**0.009221**	0.068372	0.074015	−0.045001	0.063270	−0.086218	**0.008104**	0.068334	0.075644	−0.031236	0.062662	−0.073170	−0.011120	−0.019930
	MSE	0.020388	**0.000718**	0.009075	0.020190	0.140881	0.008171	0.208962	**0.000642**	0.007490	0.017339	0.117756	0.008243	0.190189	0.001177	0.000858
	MAE	0.099718	**0.011255**	0.073655	0.099071	0.138513	0.067489	0.184734	**0.010266**	0.072735	0.097025	0.124287	0.067656	0.170615	0.024973	0.023359
		Over-identified Case														
DGP I	Mean	0.971423	0.907334	0.955125	0.966771	0.923309	0.953181	0.927752	0.907392	0.955498	0.969082	0.921761	0.953352	0.926929	–	–
	Median	1.000000	0.900018	0.988313	0.999078	0.900245	0.983957	0.901254	0.900019	0.989163	0.999187	0.900253	0.984206	0.901551	–	–
	Bias	0.071423	**0.007334**	0.055125	0.066771	0.023309	0.053181	0.027752	**0.007392**	0.055498	0.069082	0.021761	0.053352	0.026929	–	–
	MSE	0.032857	**0.000579**	0.005692	0.021110	0.016934	0.005429	0.017052	**0.000582**	0.005694	0.017261	0.017468	0.005416	0.018445	–	–
	MAE	0.110686	**0.008741**	0.057917	0.091781	0.049096	0.055535	0.050273	**0.008743**	0.058081	0.089548	0.049865	0.055515	0.051434	–	–
DGP II	Mean	0.984962	0.905206	0.961616	0.974132	0.898238	0.957846	0.910704	0.905271	0.961614	0.970946	0.891291	0.957539	0.906623	–	–
	Median	1.000000	0.900014	0.992815	0.998447	0.930982	0.989510	0.935006	0.900012	0.993100	0.998875	0.926941	0.989264	0.935006	–	–
	Bias	0.084962	**0.005206**	0.061616	0.074132	−0.001762	0.057846	0.010704	**0.005271**	0.061614	0.070946	−0.008709	0.057539	0.006623	–	–
	MSE	0.015531	**0.000503**	0.006219	0.011436	0.064198	0.005920	0.050394	**0.000495**	0.006357	0.015586	0.072985	0.005999	0.055186	–	–
	MAE	0.099427	**0.007250**	0.064257	0.085275	0.090168	0.060907	0.079972	**0.007173**	0.064724	0.089913	0.095598	0.061396	0.083465	–	–
DGP III	Mean	0.979996	0.908979	0.960402	0.975061	0.928549	0.956217	0.938197	0.908497	0.960488	0.975037	0.927877	0.955824	0.937631	–	–
	Median	1.000000	0.900032	0.992275	0.999567	0.913514	0.988456	0.928359	0.900028	0.992414	0.999541	0.912103	0.986951	0.928539	–	–
	Bias	0.079996	**0.008979**	0.060402	0.075061	0.028549	0.056217	0.038197	**0.008497**	0.060488	0.075037	0.027877	0.055824	0.037631	–	–
	MSE	0.019546	**0.000743**	0.005994	0.012375	0.014513	0.005457	0.009831	**0.000704**	0.006001	0.011825	0.013927	0.005418	0.010165	–	–
	MAE	0.102501	**0.010903**	0.062288	0.087863	0.054327	0.057096	0.051131	**0.010423**	0.062382	0.087803	0.054147	0.056790	0.051730	–	–
DGP IV	Mean	0.981881	0.906336	0.963176	0.972500	0.886856	0.957106	0.895314	0.906244	0.961497	0.970139	0.887712	0.956900	0.896953	–	–
	Median	1.000000	0.900020	0.994575	0.999072	0.940234	0.991419	0.939497	0.900022	0.993835	0.999204	0.932461	0.990061	0.935243	–	–
	Bias	0.081881	0.006330	0.063176	0.072500	−0.013144	0.057106	**−0.004686**	0.006244	0.061497	0.070139	−0.012288	0.056900	**−0.003047**	–	–
	MSE	0.019329	**0.000571**	0.007360	0.014045	0.084768	0.007660	0.069426	**0.000520**	0.008019	0.018071	0.078494	0.006440	0.066003	–	–
	MAE	0.102318	**0.008507**	0.067331	0.087282	0.105202	0.063787	0.097084	**0.008102**	0.068038	0.090569	0.101745	0.062860	0.094515	–	–

Notes: True value: ρ=0.9. MSE = mean squared error. MAE = mean absolute error. Best performances highlighted in bold.

**Table 8:** Statistics of $\hat{\sigma }$.

	$\hat{\sigma }$	GMM	EL	ET	CUE	ETEL	HD	ETHD	SEL	SET	SCUE	SETEL	SHD	SETHD	ML	BI
		Just-Identified Case														
DGP I	Mean	0.003810	0.006412	0.004104	0.004245	0.018494	0.004453	0.017820	0.006408	0.004076	0.004246	0.018021	0.004495	0.017997	0.008057	0.006648
	Median	0.004663	0.006949	0.004769	0.004748	0.006991	0.005109	0.006979	0.006949	0.004760	0.004759	0.006992	0.005130	0.006982	0.006863	0.006621
	Bias	−0.003190	−0.000588	−0.002896	−0.002755	0.011494	−0.002547	0.010820	−0.000592	−0.002924	−0.002754	0.011021	−0.002505	0.010997	0.001057	**−0.000352**
	MSE	0.000026	0.000004	0.000020	0.000020	0.017209	0.000020	0.016273	0.000005	0.000020	0.000020	0.015871	0.000020	0.016284	0.000013	**0.000000**
	MAE	0.003670	0.000904	0.003283	0.003270	0.014925	0.003024	0.014594	0.000910	0.003291	0.003246	0.014387	0.003016	0.014745	0.002164	**0.000451**
DGP II	Mean	0.007011	0.007122	0.005744	0.006869	0.027836	0.005742	0.020027	0.007111	0.005821	0.006797	0.035098	0.005889	0.021075	0.012112	0.009219
	Median	0.006827	0.007000	0.006928	0.006832	0.007000	0.006970	0.006997	0.007000	0.006942	0.006837	0.007000	0.006971	0.006998	0.012059	0.009062
	Bias	0.000011	**0.000122**	−0.001256	−0.000131	0.020836	−0.001258	0.013027	**0.000111**	−0.001179	−0.000203	0.028098	−0.001111	0.014075	0.005112	0.002219
	MSE	0.000010	**0.000002**	0.000016	0.000019	0.027185	0.000016	0.020234	**0.000002**	0.000017	0.000014	0.040630	0.000019	0.020671	0.000048	0.000006
	MAE	0.001267	**0.000638**	0.002563	0.001665	0.024443	0.002591	0.016931	**0.000656**	0.002621	0.001658	0.031609	0.002709	0.017945	0.005135	0.002219
DGP III	Mean	0.004237	0.006413	0.004107	0.004577	0.020065	0.004313	0.01824	0.006436	0.004104	0.004609	0.017441	0.004335	0.019308	0.008654	0.007018
	Median	0.005104	0.006954	0.004953	0.005102	0.006988	0.005243	0.006968	0.006959	0.004947	0.005108	0.006989	0.005274	0.006970	0.007645	0.007024
	Bias	−0.002763	−0.000587	−0.002893	−0.002423	0.013065	−0.002687	0.011240	−0.000564	−0.002896	−0.002391	0.010441	−0.002665	0.012308	0.001654	**0.000018**
	MSE	0.000017	0.000003	0.000021	0.000017	0.019542	0.000019	0.017779	0.000003	0.000021	0.000017	0.014448	0.000019	0.019712	0.000019	**0.000000**
	MAE	0.002999	0.000802	0.003292	0.002905	0.016525	0.003060	0.015196	0.000789	0.003308	0.002886	0.013876	0.003053	0.016259	0.002397	**0.000358**
DGP IV	Mean	0.006987	0.007145	0.005701	0.006995	0.021887	0.005697	0.017672	0.007121	0.005708	0.006927	0.025074	0.005787	0.017221	0.012070	0.009144
	Median	0.006846	0.007000	0.006930	0.006872	0.007000	0.006967	0.007000	0.007000	0.006907	0.006885	0.007000	0.006980	0.007000	0.012140	0.009071
	Bias	−0.000013	0.000145	−0.001299	**−0.000005**	0.014887	−0.001303	0.010672	0.000121	−0.001292	−0.000073	0.018074	−0.001213	0.010221	0.005070	0.002144
	MSE	0.000021	**0.000003**	0.000017	0.000032	0.023086	0.000018	0.017269	**0.000003**	0.000014	0.000026	0.028071	0.000018	0.016581	0.000055	0.000005
	MAE	0.001266	**0.000671**	0.002529	0.001641	0.018523	0.002535	0.014474	**0.000692**	0.002412	0.001604	0.021612	0.002600	0.013984	0.005082	0.002144
		Over-identified Case														
DGP I	Mean	0.005106	0.006275	0.004553	0.004665	0.020639	0.004451	0.017078	0.006279	0.004540	0.004593	0.020817	0.004459	0.016549	–	–
	Median	0.004763	0.006932	0.005216	0.004805	0.006981	0.005185	0.006981	0.006935	0.005210	0.004800	0.006984	0.005200	0.006983	–	–
	Bias	−0.001894	−0.000725	−0.002447	−0.002335	0.013639	−0.002549	0.010078	−0.000721	−0.002460	−0.002407	0.013817	−0.002541	0.009549	–	–
	MSE	0.000018	0.000003	0.000015	0.000024	0.017406	0.000016	0.012898	0.000003	0.000015	0.000020	0.016300	0.000016	0.011297	–	–
	MAE	0.002544	0.000844	0.002719	0.003029	0.016779	0.002834	0.013127	0.000837	0.002758	0.002966	0.016854	0.002841	0.012563	–	–
DGP II	Mean	0.006964	0.007104	0.006187	0.006851	0.028620	0.006100	0.021861	0.007102	0.006228	0.007027	0.032394	0.006130	0.024782	–	–
	Median	0.006704	0.007000	0.006994	0.006858	0.007000	0.006995	0.007000	0.007000	0.006992	0.006859	0.007000	0.006995	0.006999	–	–
	Bias	**−0.000036**	0.000104	−0.000813	−0.000149	0.021620	−0.000900	0.014861	0.000102	−0.000772	0.000027	0.025394	−0.000870	0.017782	–	–
	MSE	0.000008	**0.000002**	0.000012	0.000011	0.031451	0.000014	0.021158	**0.000002**	0.000013	0.000023	0.038390	0.000014	0.026533	–	–
	MAE	0.001091	**0.000562**	0.002035	0.001488	0.024862	0.002199	0.018187	**0.000557**	0.002138	0.001644	0.028764	0.002284	0.021316	–	–
DGP III	Mean	0.005304	0.006376	0.004549	0.004785	0.020496	0.004729	0.015126	0.006389	0.004582	0.004782	0.018792	0.004743	0.013350	–	–
	Median	0.005089	0.006949	0.005410	0.005162	0.006988	0.005595	0.006982	0.006949	0.005430	0.005163	0.006989	0.005598	0.006983	–	–
	Bias	−0.001696	−0.000624	−0.002451	−0.002215	0.013496	−0.002271	0.008126	−0.000611	−0.002418	−0.002218	0.011792	−0.002257	0.006350	–	–
	MSE	0.000010	0.000002	0.000015	0.000013	0.018838	0.000014	0.011542	0.000002	0.000015	0.000012	0.014532	0.000014	0.007820	–	–
	MAE	0.002098	0.000740	0.002732	0.002550	0.016568	0.002594	0.011308	0.000736	0.002726	0.002538	0.014832	0.002570	0.009542	–	–
DGP IV	Mean	0.007122	0.007073	0.006165	0.006803	0.035439	0.006127	0.015132	0.007093	0.006184	0.006956	0.038059	0.006052	0.023901	–	–
	Median	0.006817	0.007000	0.006996	0.006869	0.007000	0.007000	0.007000	0.007000	0.006997	0.006886	0.007000	0.006999	0.007000	–	–
	Bias	0.000122	0.000073	−0.000835	−0.000197	0.028439	−0.000873	0.008132	0.000093	−0.000816	**−0.000044**	0.031059	−0.000948	0.016901	–	–
	MSE	0.000012	**0.000002**	0.000012	0.000014	0.040050	0.000014	0.009311	**0.000002**	0.000014	0.000020	0.046360	0.000013	0.024994	–	–
	MAE	0.001075	**0.000548**	0.001931	0.001405	0.031775	0.002118	0.011626	**0.000561**	0.002087	0.001464	0.034464	0.002218	0.020501	–	–

Notes: True value: σ=0.007. MSE = mean squared error. MAE = mean absolute error. Best performances highlighted in bold.

## Data Availability

The data used were simulated in a Monte Carlo study.

## Appendix A. Moment Conditions

From the first-order conditions (5) and (6), we derive the first two moment conditions:

(A1) $$\begin{array}{c} g_{1}\left(x_{t},\theta \right)\equiv b-w_{t}c_{t}^{-\gamma } \end{array}$$

(A2) $$\begin{array}{c} g_{2}\left(x_{t},\theta \right)\equiv \beta \left(1-\delta +r_{t+1}\right){\left(\frac{c_{t+1}}{c_{t}}\right)}^{-\gamma }-1. \end{array}$$

Applying the expectation operator on the technology level Equation (2), we obtain

(A3) $$E[\ln z_{t}-\rho \ln z_{t-1}]=0$$

and using $\varepsilon_{t}∼\left(0,\sigma^{2}\right)$, we know that

(A4) $$E[{\left(\ln z_{t}-\rho \ln z_{t-1}\right)}^{2}-\sigma^{2}]=0.$$

Although $z_{t}$ is an unobservable variable, we can use the production function defined in (1) to put the technological level as a function of product $y_{t}$, capital stock $k_{t}$, and hours worked $n_{t}$:

(A5) $$\begin{array}{c} y_{t}=z_{t}k_{t}^{\alpha }n_{t}^{1-\alpha }\;\Rightarrow \;z_{t}=y_{t}k_{t}^{-\alpha }n_{t}^{-(1-\alpha )}\;\Rightarrow \;\ln z_{t}=\ln y_{t}-\alpha \ln k_{t}-\left(1-\alpha \right)\ln n_{t} \end{array}$$

We defined two new moment conditions combining (A3) and (A4) with (A5):

(A6) $$\begin{array}{c} g_{3}\left(x_{t},\theta \right)\equiv \ln y_{t}-\rho \ln y_{t-1}-\alpha \left(\ln k_{t}-\rho \ln k_{t-1}\right)-\left(1-\alpha \right)\left(\ln n_{t}-\rho \ln n_{t-1}\right) \end{array}$$

(A7) $$\begin{array}{c} g_{4}\left(x_{t},\theta \right)\equiv {\left[\ln y_{t}-\rho \ln y_{t-1}-\alpha \left(\ln k_{t}-\rho \ln k_{t-1}\right)-\left(1-\alpha \right)\left(\ln n_{t}-\rho \ln n_{t-1}\right)\right]}^{2}-\sigma^{2} \end{array}$$

Since parameter α is calibrated, moment conditions (A6) and (A7) identify ρ and σ. However, with four moment conditions generated from model equations, we are not yet able to identify β, γ, and *b*. In this way, we will have to resort to the definition of artificial moment conditions, as in [7]. Using original moment conditions (A1) and (A2), we write the following three artificial moment conditions:

(A8) $$\begin{array}{c} g_{5}\left(x_{t},\theta \right)\equiv {\left(g_{1}\left(x_{t},\theta \right)\right)}^{2}⊙{\left(g_{2}\left(x_{t},\theta \right)\right)}^{2} \end{array}$$

(A9) $$\begin{array}{c} g_{6}\left(x_{t},\theta \right)\equiv {\left(g_{1}\left(x_{t},\theta \right)\right)}^{2}⊙g_{2}\left(x_{t},\theta \right) \end{array}$$

(A10) $$\begin{array}{c} g_{7}\left(x_{t},\theta \right)\equiv g_{1}\left(x_{t},\theta \right)⊙{\left(g_{2}\left(x_{t},\theta \right)\right)}^{2} \end{array}$$

where ⊙ is a Hadamard product.

## Appendix B. Local and Global Misspecification in Econometric Models

Misspecification in econometric models occurs when the assumed model differs from the true data-generating process (DGP). This discrepancy can arise from incorrect functional forms, omitted variables, improper distributional assumptions, or errors in specifying moment conditions. The impact of misspecification on estimators depends on its nature, which can be broadly classified into *local* and *global* misspecification. Understanding these concepts is crucial for evaluating the robustness of estimators, i.e., their ability to deliver reliable results despite such discrepancies.

Local misspecification refers to small, asymptotically negligible deviations from the assumed model. These deviations are often treated as perturbations that do not drastically alter the asymptotic properties of estimators. Formally, if the true moment condition is

E[g(X,θ)]=0,

then a locally misspecified model assumes

$$E\left[g\left(X,\theta \right)\right]=\delta_{n},$$

where $\delta_{n}\to 0$ as the sample size n→∞. Such deviations vanish asymptotically, allowing estimators to remain consistent under suitable regularity conditions.

Examples of local misspecification include minor inaccuracies in specifying transition probabilities or utility functions in dynamic models. Estimators such as the generalized method of moments (GMM) and generalized empirical likelihood (GEL) methods often exhibit robustness to local misspecification. For instance, smoothing techniques used in GEL estimators can handle perturbations in moment conditions, ensuring consistent and efficient parameter estimates.

Global misspecification refers to significant deviations where the assumed model structure fundamentally differs from the true DGP. In this case, the moment conditions are violated, such that

E[g(X,θ)]=δ,

where δ≠0 and does not vanish as n→∞. Global misspecification typically leads to biased and inconsistent estimators because the foundational assumptions required for estimation are invalid.

An example of global misspecification is the application of a linear regression model to data with an inherently nonlinear relationship. Such structural errors require adopting more flexible modeling approaches, such as semiparametric or nonparametric methods, to accurately capture the underlying data dynamics.

The robustness of an estimator refers to its ability to produce reliable and consistent results despite potential model misspecification. Estimators robust to local misspecification maintain their consistency and efficiency despite small deviations in the assumed model. For example, GMM estimators adapt to local perturbations in the moment conditions through their weighting matrix, preserving asymptotic efficiency. GEL and GMC methods, such as the smoothed empirical likelihood (SEL), achieve robustness by incorporating smoothing techniques, which reduce the sensitivity to minor discrepancies in the moment conditions.

Global misspecification poses a greater challenge, as it often results in bias and inconsistency. Estimators robust to global misspecification rely on minimizing strict parametric assumptions. For instance, quasi-maximum likelihood estimators (QMLEs) are robust to certain forms of global misspecification, as they rely on the first two moments of the data rather than full distributional assumptions. Over-identified GMM estimators can detect global misspecification through overidentifying restriction tests. However, if the misspecification persists, the resulting estimates may still be biased. For a detailed discussion of misspecification problems in econometric models and moment-based estimators, see [37,65,66,67]. For an analysis of these issues specifically in the context of rational expectations models, refer to [68].
